# Glycosylated modification of MUC1 maybe a new target to promote drug sensitivity and efficacy for breast cancer chemotherapy

**DOI:** 10.1038/s41419-022-05110-2

**Published:** 2022-08-16

**Authors:** Xiaomin Xi, Jiting Wang, Yue Qin, Weidong Huang, Yilin You, Jicheng Zhan

**Affiliations:** Beijing Key Laboratory of Viticulture and Enology, College of Food Science and Nutritional Engineering, China Agricultural University, Beijing, People’s Republic of China

**Keywords:** Breast cancer

## Abstract

Breast cancer, the most common cancer in women, usually exhibits intrinsic insensitivity to drugs, even without drug resistance. MUC1 is a highly glycosylated transmembrane protein, overexpressed in breast cancer, contributing to tumorigenesis and worse prognosis. However, the molecular mechanism between MUC1 and drug sensitivity still remains unclear. Here, natural flavonoid apigenin was used as objective due to the antitumor activity and wide availability. MUC1 knockout (KO) markedly sensitized breast cancer cells to apigenin cytotoxicity in vitro and in vivo. Both genetical and pharmacological inhibition significantly enhanced the chemosensitivity to apigenin and clinical drugs whereas MUC1 overexpression conversely aggravated such drug resistance. Constitutively re-expressing wild type MUC1 in KO cells restored the drug resistance; however, the transmembrane domain deletant could not rescue the phenotype. Notably, further investigation discovered that membrane-dependent drug resistance relied on the extracellular glycosylated modification since removing O-glycosylation via inhibitor, enzyme digestion, or GCNT3 (MUC1 related O-glycosyltransferase) knockout markedly reinvigorated the chemosensitivity in WT cells, but had no effect on KO cells. Conversely, inserting O-glycosylated sites to MUC1-N increased the drug tolerance whereas the O-glycosylated deletant (Ser/Thr to Ala) maintained high susceptibility to drugs. Importantly, the intracellular concentration of apigenin measured by UPLC and fluorescence distribution firmly revealed the increased drug permeation in MUC1 KO and BAG-pretreated cells. Multiple clinical chemotherapeutics with small molecular were tested and obtained the similar conclusion. Our findings uncover a critical role of the extracellular O-glycosylation of MUC1-N in weakening drug sensitivity through acting as a barrier, highlighting a new perspective that targeting MUC1 O-glycosylation has great potential to promote drug sensitivity and efficacy.

## Introduction

Breast cancer is the most frequently diagnosed cancer and the second leading cause of mortality and accounts for estimated 30% of tumors diagnosed among women worldwide[1]. It is a highly heterogeneous disease with different clinical manifestations and multiple signaling pathways mediate its initiation and progression [2]. Based on genetic patterns and molecular portraits, breast cancer has been classified into four subtypes including Luminal A, Luminal B, HER2-enriched, and triple-negative (TNBC). The current treatment regimens include mastectomy, chemotherapy, radiotherapy, endocrine therapy, and novel immunotherapy, which is treated based on individual clinical characteristics and tumor grade[3]. Although progress in the early detection, diagnosis, and treatment of breast cancer has been achieved in recent decades, intrinsic insensitivity or acquired drug resistance, especially to chemotherapy, has become a daunting challenge in the clinical treatment. Trying to re-sensitize cancer cells to drugs is one of the most valuable tasks for present [4].

The oncogenic MUC1 has been found overexpressed in breast cancer and commonly predicts worse prognosis in patients [5, 6]. MUC1 contains two heterodimers, MUC1-N (N-terminal) and MUC1-C (C-terminal). MUC1-C that few O-glycosylated is divisible into extracellular, intracellular, and cytoplasmic domain, which has been widely studied for decades [7]. MUC1 is a well-known transmembrane glycoprotein that highly glycosylated due to the presence of a variable number of tandem repeats (TR) and irregular tandem repeats (USTR) that contain rich O-linked glycosylation sites on MUC1-N. The heavily glycosylated extracellular domain extends up to 200–500 nm from the cell surface, frequently terminated with abundant neuraminic (sialic) acids [8, 9]. In details, MUC1-N is extensively O-glycosylated but moderately N-glycosylated. O-glycosylation contributes to 50–90% of the total weight of MUC1 based on the number of TR and the expression of related glycosyltransferases [10]. The canonical mucin type O-glycosylation is initiated via N‑acetylgalactosamine (GalNAc), the first monosaccharide that connects serine or threonine, which can be extended into various different structures [11, 12]. Normally, the mucinous gel made of dense carbohydrate protects the underlying epithelia from pollutants, changes in pH, desiccation, or microorganism [13]. The extended sugar branches with negative charge create a physical barrier to block the external substances and impart an antiadhesive property, limiting accessibility and preventing pathogenic colonization [14]. However, the aberrantly O-glycosylation provides MUC1 with oncogenic biological properties to promote tumor progression, anoikis escape, and metastasis [7, 15–17]. Furthermore, glycosylated MUC1 is associated with higher tumor grades and poor prognosis [18]. Recent studies have outlined an increase in O-glycans expressing sialylation noticed to facilitate metastasis in MCF-7 cells [19, 20]. Previous studies mainly focused on MUC1-C mediated downstream signaling pathways; however, few studies explored the molecular mechanism of MUC1-N, especially in drug response and efficacy.

Natural products are essential sources for prevention and treatment of diseases. The drugs used in various traditional medicines in the world almost belong to natural sources. According to a review, about 61% of the 877 new small chemical drugs introduced between 1981 and 2002 were derived from natural products or its derivatives [21]. In this study, we used apigenin as objective, a traditional flavone medicine extracted from herbs with long term of administration. Expect for other bioactive functions, apigenin exerts excellent proliferation inhibitory effect on multiple cancer cell lines [22]. Importantly, it has minimal side effects on normal cells but with economic price and accessible features. It widely exists in daily diet like onions, grapefruits, oranges, parsley, and chamomile, facilitating it has great potential for daily uptake [23]. Unluckily, the bioavailability and absorption of apigenin is particularly limited, making it difficult to achieve the anticipated effects in small dose. Hence, new strategy to improve the sensitivity to natural medicine is urgently needed.

In this study, we found MUC1 deficiency dramatically enhanced the drug efficacy in vitro and in vivo. Molecular biology revealed that only the membrane-bound MUC1 conferred the resistance to drugs whereas the transmembrane or O-glycosylation deletant mutants lost such function. Importantly, we systematically proved that the O-glycosylation of MUC1-N was responsible for MUC1 induced drug resistance by reducing intracellular drug uptake. Finally, we analyzed the possible effects of MUC1-N and MUC1-C on cell survival with or without drug treatment. Our research will deepen the understanding of poor cellular bioavailability of small molecular drugs and provide a novel insight to overcome intrinsic drug resistance in breast cancer cells.

## Results

### Targeting MUC1 sensitized breast cancer cells to drugs and enhanced drug efficacy

**Fig. 1:**
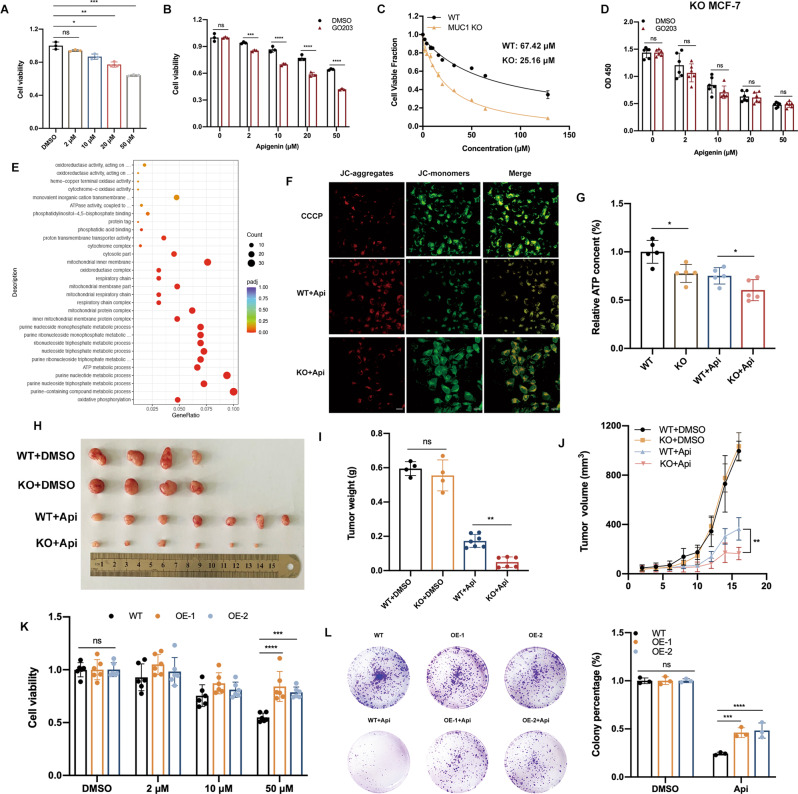
MUC1 KO enhanced the cytotoxic sensitivity to apigenin in MCF-7 cells. **A** Cell viability of WT MCF-7 cells treated with apigenin for 48 h. **B** Cell viability of WT cells treated with 5 μM GO203 combined with certain concentrations of apigenin, respectively. **C** IC_50_ of WT/KO cells treated with various concentrations of apigenin for 48 h. **D** Cell viability of KO cells treated with 5 μM GO203 combined with certain concentrations of apigenin, respectively. **E** The GO analysis of WT/KO cells (downregulated signaling pathways). **F** Determination of Mitochondrial membrane potential in WT/KO cells treated with apigenin. After apigenin exposure for 48 h, the red fluorescence intensity of KO cells was downregulated, while green fluorescence intensity was upregulated compared with the WT group. These changes indicated the decreased membrane potential. **G** Relative ATP content of WT/KO cells with/without apigenin treatment. **H** Xenograft images were shown. WT/KO MCF-7 cells seeded female BALB/c mice were treated with drug solvent or 40 mg/kg/d (i.p.) of apigenin for 16 days. **I** Tumor weight was shown. **J** Tumor size was shown. Mean ± SD. **K** Cell viability of MUC1 overexpressing MCF-7 cell lines treated with apigenin for 48 h. **L** Colony formation of MUC1 overexpressing MCF-7 cell lines treated with 25 μM apigenin for a week. Each group was analyzed in triplicate. *P < 0.05; **P < 0.01; ***P < 0.001 for comparisons.

Previous studies have showed that apigenin inhibited the proliferation and metastasis of breast cancer cells; however, such inhibitory effect was not effective enough since large dose of apigenin was quite needed, which was the main reason for limited application of natural drugs [24]. Therefore, we wondered whether loss of MUC1 could enhance the sensitivity to apigenin in breast cancer cells. As shown in Fig. S1A–D, MUC1 is amplified in human breast tumor tissues and its overexpression was associated with worse prognosis. Apigenin presented an inhibitory effect on MCF-7 cells but more than 60% cells were still alive at 50 μM concentration for 48 h (Fig. 1A). To unravel the potential role of MUC1 in drug resistance, we used MUC1 inhibitor GO203 to reduce endogenous MUC1 expression [25]. The proper concentration of GO203 was selected to eliminate extra cell damage (Fig. S2G). Interestingly, GO203 pretreatment significantly promoted the cell death compared with single apigenin group (Fig. 1B). To further explore the underlying function of MUC1 in drug chemosensitivity, CRISPR Cas9 mediated MUC1 knockout (KO) cells were constructed and confirmed by qPCR and western blot (Fig. S2A, B). No obvious viability difference was observed between wild type (WT) and KO cells, suggesting that MUC1 deletion is not lethal (Fig. S2C). Surprisingly, the IC_50_ (half maximal inhibitory concentration) of apigenin in KO cells was efficiently reduced from 67.42 μM to 25.16 μM with more than twice fold sensitization (Fig. 1C). Besides, MUC1 KO significantly prompted the apigenin induced cell death measured by cell viability and colony formation, and the living cells of KO group just remained about 20% at 50 μM apigenin, which was one third of surviving WT cells (Fig. S2D–F). Nevertheless, KO cells with GO203 pretreatment did not trigger more apigenin induced cell death, indicating that the drug efficacy was implicated in MUC1 expression (Fig. 1D). Additionally, colony formation assay was in line with above results (Fig. S2H).

To obtain an indication of discrepancies between WT and KO cells, we compared quantitative mRNA profiles of cells treated with or without apigenin. Obviously, biological functions on oxidative phosphorylation were downregulated in KO cells treated with apigenin, especially the mitochondria respiratory chain (MRC) (Fig. 1E). Multiple genes composed of mitochondrial oxidative phosphorylation enzyme complexes were detected and the results were consistent with RNA-Seq results (Fig. S6A). Importantly, apigenin markedly strengthened such vulnerability in KO cells, hinting the enhanced drug efficacy (Fig. S6B). Using CCCP as positive control, apigenin induced less JC-aggregates but more JC monomers in KO cells, illustrating stronger mitochondrial membrane potential (MMP) collapse that serves as an indicator for impaired mitochondrial (Fig. 1F). Subsequently, the relative ATP content was sharply reduced in apigenin treated KO cells due to the impaired mitochondrial integrity (Fig. 1G). Most importantly, such observation was recapitulated in mouse xenograft assay. The mice seeded with KO cells obviously had better sensitivity to apigenin, whose tumor volume and weight was robustly downregulated (Fig. 1H–J). To further identify the role of MUC1 involved in apigenin sensitivity, MUC1 was overexpressed in MCF-7 cells then confirmed (Fig. S2I). As expected, the overexpressing cells (OE) displayed significant reduced sensitivity to apigenin especially under high doses of apigenin (Figs. 1K, L, S2J). Taken together, these results strongly indicated that targeting MUC1 markedly enhanced the drug sensitivity and cellular efficacy.

### MUC1 inhibition enhanced the apigenin induced apoptosis and cell cycle arrest

**Fig. 2:**
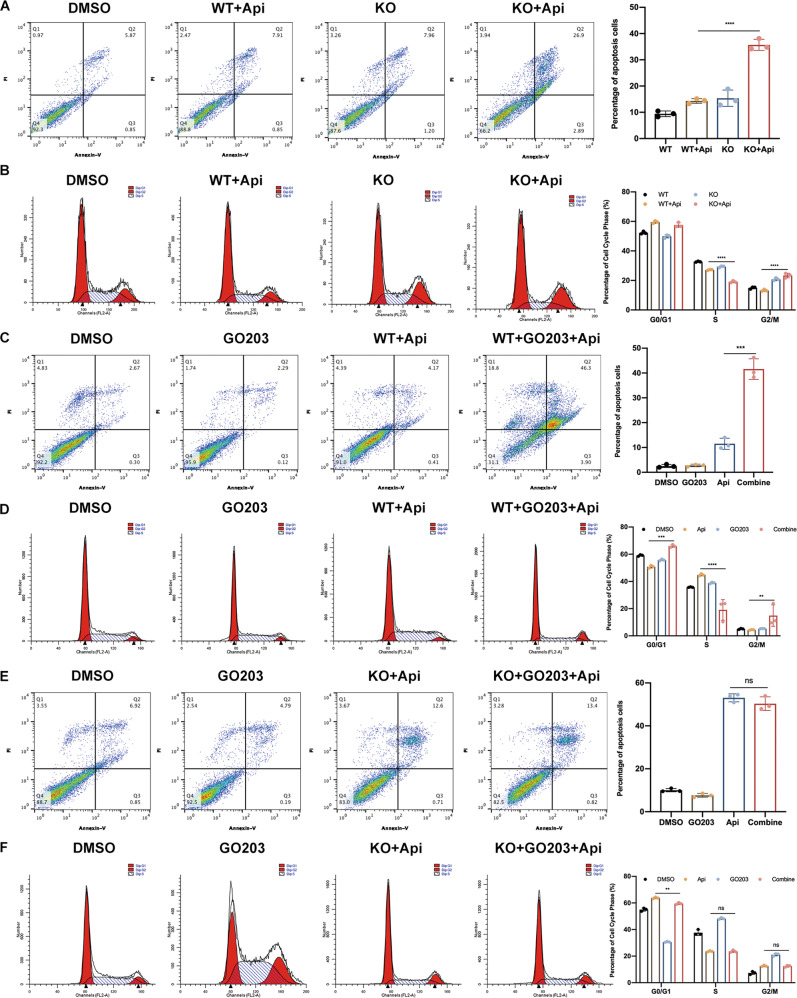
Targeting MUC1 enhanced the apoptosis and cell cycle arrest in MCF-7 cells. **A** Apoptotic flow cytometry of WT/KO cells treated with 50 μM apigenin for 48 h. **B** Cell cycle flow cytometry of WT/KO MCF-7 cells treated with 50 μM apigenin for 48 h. **C** Apoptotic flow cytometry of WT MCF-7 cells treated with GO 203 and apigenin for 48 h. **D** Cell cycle flow cytometry of WT MCF-7 treated with GO203 and apigenin for 48 h. **E** Apoptotic flow cytometry of KO MCF-7 cells treated with GO 203 and apigenin for 48 h. **F** Cell cycle flow cytometry of KO MCF-7 treated with GO203 and apigenin for 48 h. Each group was analyzed in triplicate. *P < 0.05; **P < 0.01; ***P < 0.001 for comparisons.

To further explore the mechanism of cell death caused by increased apigenin sensitivity via MUC1 inhibition, apoptosis and cell cycle arrest were detected both in genetic and pharmacological levels. As expected, apoptosis evaluation demonstrated that apigenin improved the apoptotic percentage in KO cells as determined by flow cytometry and cleaved-PARP compared with WT groups (Figs. 2A, S2L). On terms of cell cycle, apigenin markedly blocked the transition from G2 to M phase and decreased S phase proportion in KO cells (Fig. 2B). Pharmacologically, the pretreatment of GO203 for 24 h thereafter apigenin caused graver apoptosis in WT cells, whereas such effect disappeared in KO cells, proving that GO203 promoted sensitization depended on MUC1 expression (Fig. 2C, E). Likewise, WT MCF-7 cells pretreated with GO203 and apigenin induced obvious cell cycle arrest on G2/M and downregulated S phase percentage; however, such phenotype was not observed in KO groups (Fig. 2D, F). Taken together, these results presented that the enhanced drug sensitivity in MUC1 KO cells led to the elevated apoptosis and cell cycle arrest; meanwhile, such process required MUC1 expression. Importantly, to eliminate the interference of cell model, we knocked out MUC1 in another breast cancer cell line MDA-MB-231 and replicated the experiments (Fig. S3A, B). Overall, MUC1 KO cells were significantly more sensitive to apigenin, as evidenced by down-regulation of IC_50_, cell viability and colony formation (Fig. S3C–G). Similarly, GO203 was able to induce a greater percentage of cell death in WT MDA-MB-231 with significantly increased levels of apoptosis and cell cycle arrest, but not in MUC1 KO cells (Figs. S3H–J, S4C, D, S5B, C). Genetically, the increased drug sensitivity of MUC1 deficient cells resulted in higher percentage of apoptosis and cell cycle arrest (Figs. S4A, B, S5A). Taken together, we obtained similar results in both breast cancer cell lines.

### Transmembrane domain was strongly requisite for MUC1 induced drug resistance

**Fig. 3:**
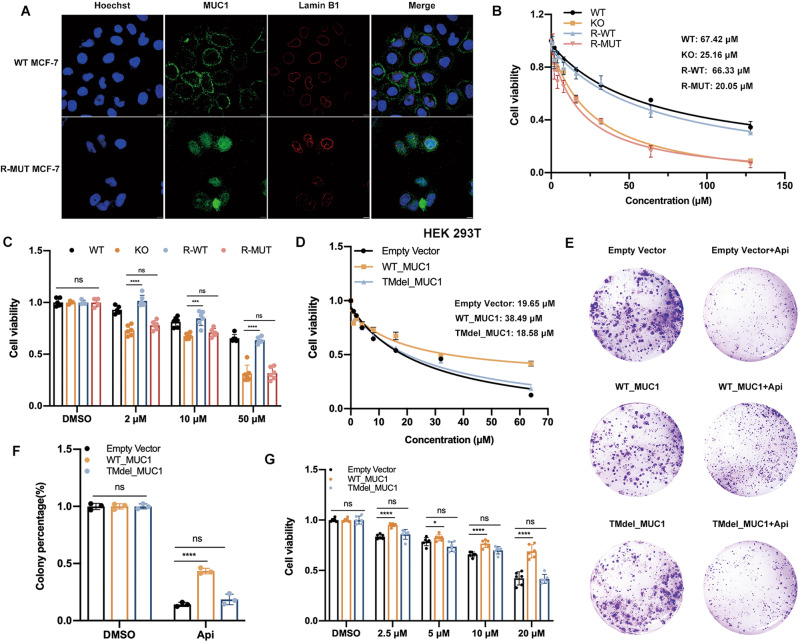
The transmembrane domain was highly required for MUC1 induced drug resistance. **A** Immunofluorescence assay using MUC1 and Lamin B1 antibody was performed to confirm the MUC1 distribution in WT and R-MUT cells. Blue: Hoechst for nuclear staining; Red: Lamin B1 for nuclear membrane staining; Green: MUC1 staining. In WT cells, MUC1 evenly distributes on cell surface; however, the mutant MUC1 gathered in the cytoplasm and cannot anchor on cell membrane due to the TM deletion. **B** IC_50_ of four cell lines (namely WT, KO, re-expressed WT MUC1 in KO cells (R-WT) and re-expressed mutant MUC1 in KO cells (R-MUT)) treated with various concentrations of apigenin for 48 h. **C** Cell viability of re-constitutively expressed MCF-7 cells treated with indicated dose of apigenin for 48 h. **D** IC_50_ of empty vector, WT_MUC1 and TMdel_MUC1 plasmids transfected 293 T cell lines treated with various concentrations of apigenin for 48 h. **E** Colony formation of empty vector, WT_MUC1 and TMdel_MUC1 plasmids transfected 293 T cell lines treated with 25 μM apigenin for a week. **F** The quantification of **E. G** Cell viability of empty vector, WT_MUC1 and TMdel_MUC1 plasmids transfected 293 T cell lines treated with indicated dose of apigenin for 48 h. Each group was analyzed in triplicate. *P < 0.05; **P < 0.01; ***P < 0.001 for comparisons.

Previous studies have showed that MUC1-C translocated to mitochondria and destroyed the BAX induced endogenous apoptosis by binding to BH3 domain. Here, we interrogated whether MUC1-N, which is extracellularly anchored on the cell membrane, contributed to drug resistance. We tested the hypothesis by adopting a strategy that does not alter the MUC1-N sequence but makes it no longer anchor on cytomembrane. Therefore, we constructed a MUC1 mutant whose transmembrane sequence (TM) was deleted but the rest functional domain kept intact, including CQC and phosphorylated sites of intracellular segment (MUC1-CT). Briefly, the plasmids expressing flag-tagged WT (WT_MUC1) or TM deleted mutant MUC1 (TMdel_MUC1) were constructed, constitutively re-expressed in MUC1 KO cells, namely R-WT and R-MUT. In fact, no significant alteration was observed on protein expression pattern between R-WT and R-MUT, implying that the general post modification of MUC1 was not affected by TM abrogation (Fig. S6E). Regarding the localization, MUC1 evenly distributed on cell membrane in WT cells; on the contrary, MUC1 in R-MUT cells mostly aggregated in the cytoplasm, particularly on the surrounding of nuclear (Fig. 3A). Further, MUC1 co-localized with flag in R-MUT cells (Fig. S6D). Then, the results of cell viability, drug IC_50_ and colony formation revealed that R-MUT cells, performing similar to KO cells, could not restore the original resistance to apigenin; whereas R-WT cells were quite efficient to rescue the phenotype with an approximate IC_50_ as WT cells, suggesting that TM domain was highly required for MUC1 functionally induced drug resistance (Figs. 3B, C, S6C). The colony formation was in line with above results (Fig. S6F).

Next, we transfected WT and TMdel mutant MUC1 plasmids with lenti-virus into MUC1 background blank HEK 293 T cell line wherein the MUC1 expression pattern was found similar as MCF-7 cells, suggesting that MUC1 was successfully expressed and maturely O-glycosylated (Fig. S2K). The results demonstrated that the HEK 293 T cells transfected with TMdel_MUC1 had no significant difference in drug sensitivity compared with empty vector cells; however, WT_MUC1 transfected cells had elevated IC_50_ and alleviated cell death (Fig. 3D, G). Meanwhile, the ability of monoclonal formation was suppressed in TMdel mutant (Fig. 3E, F). Taken together, these results provide evidence that no matter in MUC1 KO MCF-7 breast cancer cells or MUC1 absent HEK 293 T cells, the membrane relied distribution of MUC1-N intensely impacts the drug sensitivity.

### Extracellular O-glycosylation of MUC1-N directly determined the drug sensitivity

Based on above results, we have identified that MUC1 directly determined the drug sensitivity mediated through its membrane anchorage. We wondered whether the declined drug sensitivity was resulted from the combination between MUC1 and apigenin. In MUC1 KO MCF-7 cells, the addition of human recombinant MUC1 (non-glycosylated, 25 μg/ml) together with apigenin for 24 h did not attenuate the cell death compared with single apigenin groups, indicating that there was possibly no direct interaction between MUC1 and apigenin (Fig. S6G). To increase the contacting time, we cultured the cells with 10 μg/ml recombinant MUC1 and apigenin for 48 h and obtain the same results (Fig. S6H). We also reduplicated in MUC1 deficient HEK 293 T cells (data not shown). These results pointed that MUC1 induced drug resistance possibly depends on a certain mechanism demanding cell surface localization independent of protein-drug binding. Therefore, we speculated that certain structures or modifications of MUC1 on cell membrane mediated such drug resistance.

**Fig. 4:**
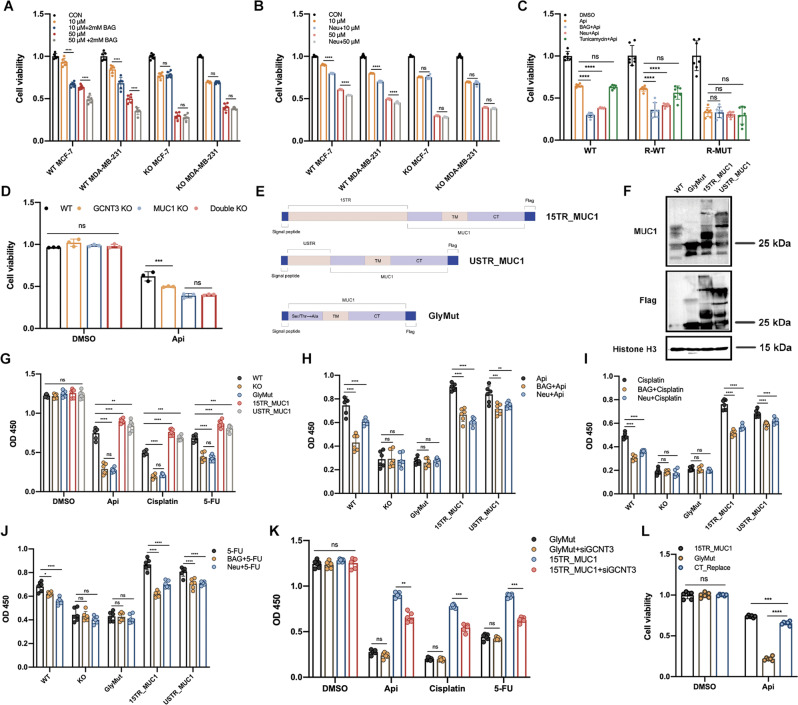
MUC1 glycosylation inhibition re-sensitized MCF-7 cells to drug cytotoxicity. **A** Cell viability of WT/KO cells pretreated with 2 mM BAG post apigenin treatment for 48 h. Both MCF-7 and MDA-MB-231 were detected. **B** Cell viability of WT/KO cells pretreated with Neu post apigenin treatment for 48 h. Both MCF-7 and MDA-MB-231 were detected. **C** Cell viability of WT, R-WT, and R-MUT cell lines pretreated with BAG, Neu or tunicamycin after 50 μM apigenin treatment for 48 h. The concentration of BAG was 2 mM and tunicamycin was 0.5 ug/ml. **D** Cell viability of four cell lines (WT, GCNT3 KO, MUC1 KO, and Double KO) treated with 50 μM apigenin for 48 h. **E** The diagram of plasmids construction. **F** Western blot verification of MUC1 O-glycosylation in WT, GlyMut, 15TR_MUC1, and USTR_MUC1 cell lines. **G** OD value of five cell lines after drug treatments. **H** OD value of five cell lines pretreated with BAG or Neu post apigenin treatment for 48 h. **I** OD value of five cell lines pretreated with BAG or Neu post cisplatin treatment for 48 h. **J** OD value of five cell lines pretreated with BAG or Neu post 5-FU treatment for 48 h. **K** Cell viability of GlyMut and 15TR_MUC1 with/without siGCNT3 treated with indicated drugs. **L** Cell viability of 15TR_MUC1, GlyMut, and CT_Replace cells treated with DMSO or 50 μM apigenin for 48 h. Each group was analyzed in triplicate. *P < 0.05; **P < 0.01; ***P < 0.001 for comparisons.

**Fig. 5:**
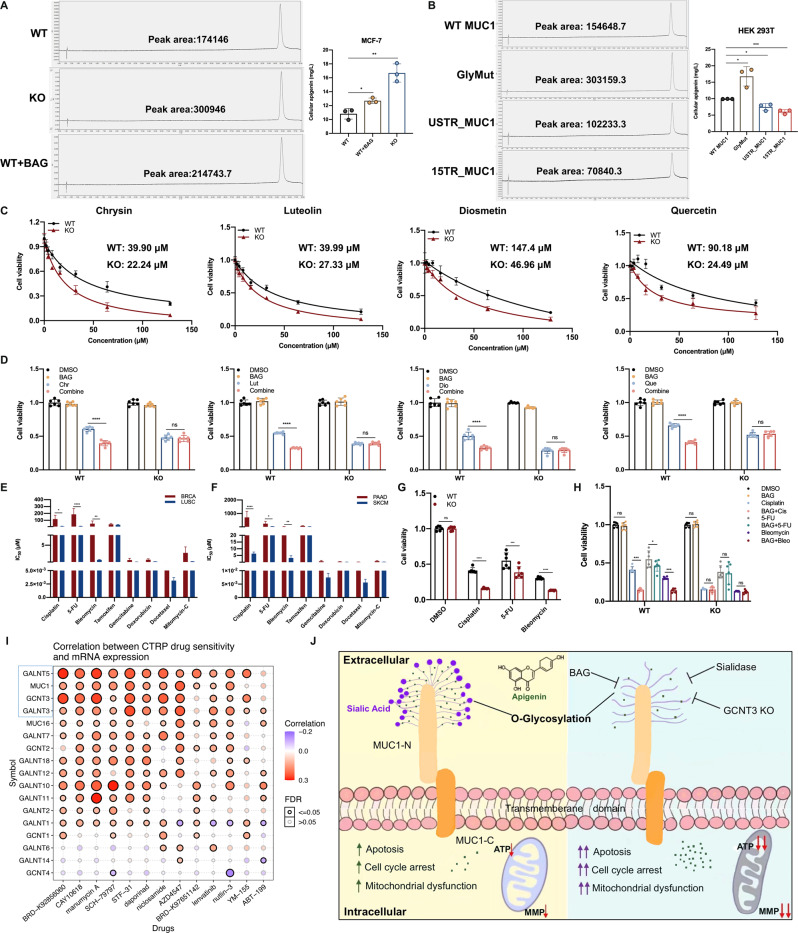
The detection of intracellular apigenin uptake and validation for multiple chemotherapeutics. **A** The determination of intracellular apigenin concentration in WT, KO and 2 mM BAG-pretreated MCF-7 cells. **B** The detection of intracellular apigenin concentration in plasmids transfected HEK 293 T cells. **C** IC_50_ of WT/KO cells treated with different natural compounds for 48 h. **D** Cell viability of WT/KO cells pretreated with 2 mM BAG for 24 h then 50 μM natural compounds for 48 h. **E** Calculated IC_50_ for selected drugs in BRCA and LUSC cell lines, respectively. MUC1 is overexpressed in BRCA, but little expressed in LUSC. **F** Calculated IC_50_ of selected drugs in PAAD and SKCM cell lines, respectively. **G** Cell viability of WT/KO cells treated with selected drugs for 48 h. **H** Cell viability of WT/KO cells pretreated with 2 mM BAG for 24 h thereafter selected drugs for 48 h. **I** Correlation between drug sensitivity and mRNA expression of MUC1 and related O-glycosyltransferase genes from CTRP database. Most of these genes were positively (red) correlated with drug tolerance, implying that MUC1 along with over-glycosylation possibly leads to drug resistance. **J** A proposed model of MUC1 O-glycosylation induced resistance to apigenin treatment. Extensive O-glycosylation on MUC1 N-terminal induces drug resistance and mitigate cellular apoptosis, cell cycle arrest and mitochondrial dysfunction. Each group was analyzed in triplicate. *P < 0.05; **P < 0.01; ***P < 0.001 for comparisons.

It has been uncovered that the external N-terminal of MUC1 is highly O-glycosylated [11]. Especially in ER positive breast cancer cells like MCF-7, the expression of O-glycosides and sialylation modification has been proved to be related with malignant metastasis [14, 15]. Herein we came up with a conjecture that extracellular O-glycosylation of MUC1-N was possibly responsible for drug response and cytotoxicity. O-glycosylation inhibitor benzyl-N-acetyl-α-galactosaminide (BAG) specifically inhibits glycosyltransferase incorporation of glucosamine into O-glycans was used [26]. Proper dose of BAG was pre-tested to exclude extra damage on cell viability (Fig. S7A). Although mRNA expression was invisibly altered (Fig. S7B), protein with high molecular was inhibited discontinuously, implying that the O-glycosylation of MUC1 was effectively suppressed by BAG (Fig. S7G). BAG pre-treated for 24 h then apigenin for another 48 h induced more serious cell death in WT cells other than MUC1 KO cells, despite of 2 mM or 4 mM BAG (Fig. 4A, S7C). Single cell suspensions were then assayed for their ability to form colonies, which was in line with cell viability (Fig. S7J). On the other hand, neuraminidase (Neu, also called sialidase) is a typical enzyme facilitating to remove N-acetyl neuraminic acids (also called sialic acids) from a variety of glycoproteins, particularly for mucins family frequently terminated with sialic acids [27]. Neu treatment for 6 h was selected to exclude the extra damage (Fig. S7D). Cells pretreated with Neu for 6 h presented moderately altered glycosylation but with no significant fold change on mRNA level (Fig. S7E, H). Similar with BAG, Neu pretreatment significantly revived the sensitization to apigenin and induced more cell death, whereas such effect was not observed in MUC1 KO cells (Fig. 4B, S7K). Furthermore, recombinant expressed R-WT/R-MUT cells were pretreated with BAG or Neu for further validation. Importantly, to identify whether N-glycosylation participates as well, tunicamycin was used as specific inhibitor[28]. Pretreatment for 24 h with 0.5 μg/ml tunicamycin did not cause extra harm on cells (Fig. S7F). Compared with single apigenin, pretreatment with BAG or NEU dramatically recovered the drug sensitivity in R-WT cells. However, such phenomenon was not observed in R-MUT cells. Interestingly, no significant difference was observed among tunicamycin treated groups that implied N-glycosylation of MUC1 possibly did not involve in such process even if MUC1-N anchors on cytomembrane (Fig. 4C). Except for endogenic monosaccharide as substrates, the extension of O-glycosylation branches relies on the coordination of a variety of glycosyltransferases like GCNT3 (Beta-1,3-galactosyl-O-glycosyl-glycoprotein), which catalyzes the formation of core 2 and core 4 O-glycan branches after core 1 O-glycan disaccharide structure, two most important steps in O-glycosylation embranchment [29]. Coincidentally, we found the expression of GCNT3 and MUC1 were positively correlated with drug resistance in subsequent database analysis (Fig. 5I). Therefore, GCNT3 was knocked out in WT and MUC1 KO cells to explore whether GCNT3 mediated O-glycan branching was relevant to MUC1-induced drug resistance (Fig. S7I, L). Not surprisingly, GCNT3 KO suppressed MUC1 O-glycosylation and restored the drug sensitivity in WT cells instead of MUC1 KO cells (Fig. 4D). Taken together, these data showed that MUC1 conferred drug resistance was dependent upon the extracellular O-glycosylation, which highly requires a certain cooperation among glycosyltransferases.

Earlier work has revealed the structure of MUC1 and almost all O-glycosylated positions concentrate on tandem repeats (TR) and USTR domain of N-terminal [30, 31]. To explore whether these domains determine MUC1-N induced resistance, we inserted fifteen TR fragments (15TR_MUC1) or single USTR fragment (USTR_MUC1) into MUC1 cDNA after the signal peptide and stably transfected the MUC1 KO cells with lentivirus. Most importantly, the O-glycosylated mutant MUC1 (GlyMut) was established by substituting all Ser/Thr (before TM domain on N-terminal) with Ala that cannot be O-glycosylation modified then transfected like above (Fig. 4E). O-glycosylation of 15TR_MUC1 and USTR_MUC1 was obviously upregulated and the molecular weight was raised up to 180 kDa from protein profile (Fig. 4F). Subsequently, we tested apigenin and two clinical chemotherapeutics cisplatin and 5-fluorouracil (5-FU) in above cell lines. Figure 4G manifested that the drug sensitivity of 15TR_MUC1 and USTR_MUC1 cells was dramatically dropped compared with WT cells but there was no significant difference between KO and GlyMut cells. Importantly, inhibiting O-glycosylation with BAG or neuraminidase re-sensitized 15TR_MUC1 and USTR_MUC1 to these agents, but not in MUC1 KO or GlyMut cells (Fig. 4H–J). The colony formation was in line with above results (Fig. S7N). Furthermore, GCNT3 was suppressed in 15TR_MUC1 and GlyMut cells with siRNA, respectively (Fig. S7M). As expected, GCNT3 knockdown partly restored the drug sensitivity in 15TR_MUC1 cells, whereas it did not work in GlyMut cells (Fig. 4K, S7O). It has been reported that MUC1 largely depends on the CQC motif to form homodimers which in turn translocates into the nucleus for oncogenic transcription [7]. To identify whether MUC1-CT (intracellular part of MUC1-C) involves in such drug resistance, the CQC motif of MUC1-CT was replaced with AQA (Cys to Ala) in 15TR_MUC1 and GlyMut plasmids respectively, namely 15TR_ΔCT and GlyMut_ΔCT. The cell survival was examined with or without 5-FU treatment. We found that the cells transfected with 15TR_MUC1 or GlyMut grew better than the corresponding mutants in the absence of 5-FU, suggesting that CQC domain indeed promotes cell growth to some extent. When treated with 5-FU, the growth rate of 15TR_MUC1 and 15TR_△CT was both inhibited but no significant difference was found (Fig. S9A). Interestingly, if the MUC1-CT of 15TR_MUC1 was replaced with GFP tag, the drug tolerance would be markedly impaired, possibly attributing to the complete elimination of cellular dimerization, phosphorylation or even protein instability (Fig. 4L). However, there was significant difference between GlyMut and GlyMut_△CT on cell survival that highlights that MUC1-CT would partly alleviate drug toxicity when glycosylation was completely deprived (Fig. S9B). Altogether, these results showed that MUC1-CT can promote cell proliferation under normal conditions, but the O-glycosylation seems to exert a more predominant role in response to external survival pressures such as chemotherapeutic agents.

### Detection of intracellular apigenin uptake and tests for multiple chemotherapeutics

We have verified that apigenin efficacy was largely decided by extracellular O-glycosylation of MUC1-N, so we raised a presumption that such heavy viscose sugar layer would reduce the cellular drug uptake by preventing agents from entering cells. Intriguingly, the intracellular apigenin concentration from KO cells detected by UPLC was significantly higher than that of WT ones, and BAG pretreatment mitigated such barrier and promoted the drug uptake to some extent (Fig. 5A). To further confirm, HEK 293 T cells were stably transfected with shown plasmids and 15TR_MUC1 had the least concentration of apigenin followed by USTR_MUC1 and WT MUC1, whereas the GlyMut had the highest concentration near to 17 mg/L (Fig. 5B). To be more intuitive, we also examined the intracellular fluorescence density of apigenin with DPBA staining assay. The distribution of apigenin was obviously increased in KO and BAG-pretreated cells compared to WT group, further confirming our conclusion (Fig. S8A). These data supported that removing MUC1 O-glycosylation markedly enhanced the effective drug penetration.

Then we wondered if apigenin analogs with similar structure would have the same phenotype. Four flavonoids were selected, namely chrysin, diosmetin, luteolin and quercetin, and the chemical structures were showed in Fig. S8C. The IC_50_ and cell viability of MUC1 KO cells treated with above compounds were markedly downregulated (Figs. 5C, S8D). BAG pretreatment induced graver cell death in WT cells instead of MUC1 KO ones (Fig. 5D). Besides, the IC_50_ of several clinical drugs with small molecular was calculated through downloading information from GDSC database (https://www.cancerrxgene.org/). Notably, BRCA (Breast invasive carcinoma) and PAAD (Pancreatic adenocarcinoma) express high level of MUC1, yet LUSC (Lung squamous cell carcinoma) and SKCM (Skin Cutaneous Melanoma) barely express MUC1 (Fig. S1A, B). Interestingly, BRCA and PAAD had generally higher IC_50_ than the other two cancer types, with significance on cisplatin, 5-FU and bleomycin, implying that BRCA and PAAD could be less sensitive to these drugs (Fig. 5E, F). To further confirm, we validated the expression level of MUC1 in two cell lines: MUC1 highly expressed pancreatic cancer cell line Capan1 and MUC1 barely expressed lung cancer cell line A549 (Fig. S8E). We tested the chemotherapeutic with significance in two cell lines and the results showed that, except for bleomycin, Capan1 was obviously more resistant to these drugs (Fig. S8F). Moreover, BAG pretreatment markedly restored the drug response in Capan1 cells, which was not found in A549 cells (Fig. S8G). We speculated that such inconsistency of bleomycin in Capan1 is possibly due to the innate genetical BRCA2 deficiency caused impaired homologous recombination (HR) since bleomycin targets to the DNA replication process, wherein high level of MUC1 cannot rescue such genotoxic death [32]. Since the data from database was the result of comprehensive simulations of multiple cell lines, our results partially reflected the tendency.

In breast cancer MCF-7 cells, the results of cell viability were basically in line with above analysis, and BAG pretreatment contributed to higher susceptibility towards these drugs that was only found in WT cells, supporting that BAG induced sensitiveness specifically relied on MUC1 expression (Fig. 5G, H). Additionally, CTRP (Cancer Therapeutics Response Portal) database was applied to detect the drug sensitivity of MUC1 and related glycosyltransferases to numerous clinical chemotherapeutics and found most of them were positively corelated with drug resistance, especially MUC1, GCNT3, and GALNT5 (Fig. 5I). Moreover, relatively high level of GCNT3 and GALNT5 was observed in tumor tissues and predicted worse prognosis in patients from GEO database (Fig. S9C, D). The proposed model was shown in Fig. 5J. Altogether, these results provide solid evidence that MUC1-N plays a critical role in modulating drug sensitivity and removing O-glycosylation formed barrier has great potential to revive drug efficacy in breast cancer cells.

## Discussion

As an essential oncogene involved in tumor progression and signaling transduction, MUC1 has been detected with high expression in numerous epithelial cancers like breast and pancreatic carcinoma [7, 33]. Indeed, previous studies discovered that MUC1-C played an important role in the development of drug resistance mediated through translocating into the nucleus to perform as chaperones of transcription factors to activate transcription and activation of carcinogenic proteins and signaling pathways like BRCA1, MDR1, and Ras-related protein [34–39]. Hence, the direct evidence involving MUC1, especially MUC1-N, that participates in drug response remains unclear. Except for stem cells, some cancer cells are inherently tolerant to clinical drugs as determined by low sensitivity and high IC_50_, not to mention those that have developed resistance to certain drugs. The functional repertoire of MUC1 has broadened significantly beyond its original carcinogenic activity in many biological processes. Our investigation into how MUC1 protects cancer cells from drug transportation provides a physiologically relevant context to understand the reason of the high expression of MUC1 in breast cancer cells.

For the first time, our finding revealed that the drug resistance induced by MUC1 highly required membrane localization. In fact, mucins are classified as two types: membrane-bound mucins and secretory mucins. The membrane-bound mucins represented as MUC1 include extracellular (N-terminal), transmembrane (TM) and intracellular (C-terminal) domains, whereas the secretory proteins represented as MUC2 are TM domain deficient due to the alternative mRNA splice [40]. Many studies focused on the phosphorylation and downstream signaling transduction of MUC1-C; however, few studies investigated the relationship between MUC1 TM domain and drug resistance [6, 41–43]. Here, we found MUC1 localization on cell surface was obligatory to drug resistance because the TM deletion mutant performs just like KO cells. We also noticed that TM deletion did not alter the MUC1 expression pattern possibly since the O-glycosylation mostly completes in Golgi before subcellular anchorage [11]. Although some secretory proteins in mucin family like Mucin 5AC have been proved to promote cancer invasion and development through driving stemness in exosomes or vesicle pathway, it is obvious that the secretory transformation of membrane-bound MUC1 was favorable to drug response [44]. Furthermore, we verified that O-glycosylation at MUC1-N appears to play a more dominating role when treated with chemotherapeutics by constructing several mutants for O-glycosylation overexpressed (15TR_MUC1) or deleted (GlyMut) plasmids. Although MUC1-CT has potential to facilitate cell proliferation, once penetrated by large amounts of drugs, the cascade activation mediated by MUC1-CT may not rescue such strong chemotoxicity. At this time, the level of extracellular O-glycosylation largely determines the cell fate acting as the first line of defense.

Typically, intestinal endothelial cells can be protected from pathogen colonization via the viscous mucus layer. Once colonized by pathogens, the MUC1-N together with pathogens will be shed and released into blood vessel to protect intestinal cells [13]. Nevertheless, it was unclear how MUC1 in breast cancer cells responds to the threat of chemotherapeutics. The expression of MUC1 in normal epithelial cells is limited to the apical surface, but covers the entire surface in many tumor cells due to the loss of polarity, thus increasing the opportunity to contact with external substances. Our results not only revealed the necessity of membrane localization of MUC1 mediated drug resistance, but also uncovered that such dependence was due to the mucin barrier formed via O-glycosylation of MUC1-N. Once the O-glycosylation was suppressed, the drug resistance would be greatly impaired albeit MUC1 on cell membrane. Inhibiting O-glycosylation of MUC1 does not affect the transport of small molecule nutrients such as glucose and glutamine, as such type of substances rely on the active transport mediated by carrier proteins instead of free diffusion. Importantly, these nutrients do not cause the shedding and release of MUC1-N as well. However, different from nutrients, stimulants such as drugs or pathogens are blocked by glycosylation of MUC1-N then irritated, shed and finally released [45, 46]. Therefore, the drugs trapped by sugar chains could be re-released into the blood vessels, further reducing cellular permeability. The effectiveness of most chemotherapeutic agents depends on adequate intracellular uptake by tumor cells. Therefore, overexpression of MUC1 with intensive O-glycosylation seriously affects drug availability.

The glycosylation process requires the participation of many glycosyltransferases and GCNT3 is a MUC1 closely related glycosyltransferase, which was overexpressed in Kras-driven mouse and human pancreatic cancer [29]. Interestingly, we noticed that GCNT4, a homologous glycosyltransferase, was upregulated in KO cells from RNA-Seq results (Fig. S8B), implying that MUC1 KO perturbed the balance of glycosylation possibly due to the compensation mechanism in organism [47]. Not surprisingly, targeting GCNT3 re-sensitizing cells to drugs and enhanced drug efficacy, consisting with previous results [29]. Earlier work pointed out that MUC1 induced drug resistance via MUC1-C upregulation of multidrug resistance genes in pancreatic cancer cells [48]. Here, we found that the loss of N-terminal O-glycosylation induced graver cell death than MUC1-C deletant, at least in drug response. Compared with 15TR_MUC1, lack of MUC1-CT mildly impaired the drug resistance possibly due to the protein instability caused by reduced dimerization or inhibited carcinogenic transcription of MUC1 [49]. Although we cannot rule out the non-specificity of BAG or neuraminidase on other glycoproteins, these results reflected O-glycosylation plays an indispensable role in drug efficacy. Most importantly, intracellular uptake of apigenin was detected by UPLC and cyto-fluorescent staining, providing the direct evidence to such mechanism. For the first time, we systematically evidenced that targeting O-glycosylation greatly augmented drug effectiveness.

Similar to untargeted chemotherapy, the efficacy of radiotherapy may also be associated with protein glycosylation. Previous evidence showed a mutually interactive relationship between these two factors. For instance, restraining N-glycosylation with tunicamycin enhanced the therapeutic efficacy of radiotherapy by suppressing the expression of carcinogenic RTKs (receptor tyrosine kinases) like EGFR, but this approach has no effect on non-transformed lung fibroblasts [50, 51]. Moreover, in addition to N-glycosylation, targeting O-glycosylation was found to overcome the inherent radio-resistance in human laryngeal carcinoma [52]. Conversely, radiation has been reported to deeply modify the glycosylation pattern like α2-3 sialylation or O-glycosylation, altering the protein profiles then further impact the efficacy of anti-tumor regimens [53]. Collectively, radiation therapy and protein glycosylation are closely correlated and regulate the functionality mutually. Therefore, removing redundant glycosylation or targeting typical glycosyltransferase before radiotherapy may be a rational and promising therapeutic strategy to tack radio-resistance.

High level of MUC1, GCNT3, and GALNT5 from database positively correlates with drug resistance, supporting our conclusion. Like MUC1, breast cancer patients with overexpressed GCNT3 and GALNT5 predict worse survival, probably due to the worse drug response mediated by over-glycosylation [54]. The content of O-glycosylation varies among people due to the different TR numbers therefore a more detailed classification is warranted to establish MUC1 O-glycosylation level as a predictive biomarker before clinical chemotherapy especially suitable for precision medical. Here, our research highlighted that in cancer cells with high level of MUC1, the referenced dosage and actual cytotoxicity of clinical drugs need to be modulated according to the level of MUC1 O-glycosylation, especially in BRCA and PAAD. Weakening O-glycosylation layer with specific inhibitor or systemically metabolic regulation before chemotherapy, or appropriately increasing the drug dose should be considered as an important strategy to counteract the limited drug response, especially for those TNBC or tumor- metastatic breast cancer patients who can only undertake chemotherapy. It is worth noting that the detection of glycosylation modification levels in clinical tumor tissue before chemotherapy could be beneficial to better assist drug administration. Differing from GO203 that interferes MUC1-C dimerization, our research highlighted a new perspective of targeting N-terminal O-glycosylation could be promising to promote drug sensitivity and efficacy, no matter for early-stage cancer patients or the ones that have developed drug resistance. Notably, such mechanism could be suitable for many carcinogenic glycoproteins distributed on cytomembrane, and the development of therapies that specifically remove carbohydrates on cancer cells should be seriously considered in future clinical trials.

## Materials and methods

### Cell culture and reagents

Human breast cancer cell lines MCF-7, MDA-MB-231 cells and human non-small cell lung cancer A549 cells were cultured in DMEM (Gibco, Invitrogen, Carlsbad, CA, USA) with 10% fetal bovine serum (FBS; Gibco, Invitrogen, Carlsbad, CA, USA), and penicillin (100 U/mL)-streptomycin (100 mg/mL). Human embryonic kidney 293 T (HEK 293 T) was grown in the same media. Human pancreatic ductal adenocarcinoma Capan1 cell line was cultured in IMDM (Gibco, Invitrogen, Carlsbad, CA, USA) with 20% fetal bovine serum (FBS; Gibco, Invitrogen, Carlsbad, CA, USA), and penicillin (100 U/mL)-streptomycin (100 mg/mL). Cells were cultured in an incubator at 37 °C with 5% CO_2_ under sterile conditions. All cells were harvested by treatment with 0.25% trypsin–ethylenediaminetetraacetic acid (Trypsin-EDTA; Gibco, Invitrogen, Carlsbad, CA, USA). Antibodies, chemotherapeutics and reagents were shown in Table S1.

### Cell viability and colony formation assay

The cell counting kit-8 (CCK-8) assay (Cat. No. C0038, Beyotime, Shanghai, China) was used to detect cell numbers as measured by OD 450. Briefly, the cells were seeded in 100 μL of complete medium at a density of 3000–4000 cells per well in a 96-well plate overnight and then treated with indicated drugs. Then, according to the CCK-8 manufacturer’s instructions, a microplate reader (Thermo Fisher, Waltham, MA, USA) was used for the absorbance detection at a wavelength of 450 nm as OD value. For colony formation assay, 1000–2000 cells were seeded in 24-well plate overnight, and treated with indicated drugs the other day, then cultured for 1–2 weeks. The cells were fixed with 4% paraformaldehyde (Cat.No. P1110, Solarbio, Beijing, China) for 15 min, then washed with PBS twice, stained with 1% crystal violet solution (Cat.No. C0121, Beyotime, Shanghai, China) for 10 min. Finally, the number of colonies were photographed and counted.

### Flow cytometry analysis

Flow cytometry analysis was performed as previously reported [55]. Apoptotic cells were assessed using FITC Annexin V Apoptosis Detection kit (Cat. No. C1062M, Beyotime, Shanghai, China) and cell cycle arrest was detected by Cell Cycle Analysis Kit with propidium staining (Cat. No. C1052, Beyotime, Shanghai, China) according to protocol.

### Real-time quantitative PCR assay

Total RNA was extracted using TRIzol reagent (Cat. No. 9108, Takara Biotechnology, Dalian, China) from cells according to the manufacturer’s instructions, and then the RNA purity and concentration at 260:280 nm was measured. After diluting RNAs to the same concentration with RNase-free water, we used HiScript III 1st Strand cDNA Synthesis Kit (Cat. No. R312, Vazyme, Nanjing, China) to reversely transcribe 1 μg total RNAs and synthesize the first-strand cDNA according to the instructions. Real-time quantitative PCR was performed using ChamQ SYBR qPCR Master Mix (Cat. No. Q341, Vazyme, Nanjing, China). All analyses were performed via CFX Connect Real-Time System (Bio- Rad, Hercules, CA, USA). GAPDH was selected as the housekeeping gene to normalize the data of each target gene. The reaction conditions were as follows: 95 °C for 30 s, followed by 35 cycles of 95 °C for 5 s, 60 °C for 30 s, and melt curve. Results were presented as the fold change relative to the control. Three independent experiments were performed. All primers were listed in Table S2.

### Western blotting

Cells were washed with cold PBS twice and the total protein of cells was extracted using RIPA Lysis buffer (Cat.No. P0013B, Beyotime, Shanghai, China) premixed with 1 mM proteinase inhibitor PMSF. The protein concentration of cell lysis was detected using a BCA protein assay kit (Cat.No. P0012S, Beyotime, Shanghai, China). Then, samples were diluted to the same concentration and denatured with 5× SDS loading buffer (Cat.No. P0280, Beyotime, Shanghai, China). Protein was subjected to 10% SDS-PAGE by electrophoresis and transferred onto a polyvinylidene fluoride (PVDF) membrane. Membranes were incubated in 5% skimmed milk for 2 h at room temperature followed by incubated with primary antibodies at 4 °C overnight. After being washed with 1× Tris-buffered saline containing Tween 20 (Cat.No. ST673, Beyotime, Shanghai, China), membranes were incubated with horseradish peroxidase (HRP)-conjugated secondary antibodies. Bands on the membrane were visualized by using BeyoECL Moon (Cat.No. P0018FS, Beyotime, Shanghai, China). For proteins of interest, band intensities were normalized to the housekeeping protein β-actin or Histone H3. All antibodies used for western blot are listed in Table S1.

### Immunofluorescence assay

Cells were fixed with 4% paraformaldehyde for 20 min and permeabilized in 0.5% Triton-X100 for 20 min then blocked with QuickBlock™ Blocking Buffer (Beyotime, Shanghai, China) for 1 h at room temperature. Cells were washed carefully with PBS for three times after each step. The fixed cells were incubated with anti-MUC1 antibody (CST, USA), anti-Flag (CST, USA) or anti-Lamin B1 (ProteinTech, Wuhan, China) for 2 h at room temperature. FITC-labeled rabbit anti-MUC1, anti-Flag or Cy3-labeled mouse anti-Lamin B1 were co-incubated with Alexa-488 or Alexa-555 secondary antibody (P0176 & P0190, Beyotime, Shanghai, China). Nuclei were stained with Hoechst 33342 (C1026, Beyotime, Shanghai, China). Finally, the cells were visualized and photographed under laser confocal microscopy (Nikon, Japan).

### ATP quantitative detection

The ATP detection kit (Cat.No. S0026, Beyotime, Shanghai, China) was used to detect the concentration of ATP according to the manufacturer’s instructions. Briefly, cells were seeded in 2 ml of complete culture medium at the same number of 1 × 10^5^ cells per well in a six-well plate overnight, and then treated with indicated drugs according to the indicated design. Remove the culture medium, wash the cells with cold PBS twice and lyse the cells with 200 μl lysate per well. In order to fully lyse the cells, pipette the cells or shake the plate repeatedly to make the lysate completely contact the cells. Usually, cells could lyse immediately when exposed to the lysate. The supernatant was obtained for subsequent determination via centrifuging at 4 °C, 12,000 g for 10 min. Standard curve and ATP detection working buffer were previously prepared according to the protocol. Add 100 μl ATP detection working buffer into the test holes of 96-well plate and place at room temperature for 5 min so that the background ATP was consumed. Then add 20 μl sample or standard into the test hole, quickly mix with micropipette, and measure RLU value with a luminometer after an interval of at least 2 s.

### Determination of mitochondrial membrane potential

The change in mitochondrial membrane potential was determined in accordance with standard procedure by using a mitochondrial membrane potential assay kit with JC-1 (Cat.No. C2006, Beyotime, Shanghai, China). Simply, WT and MUC1 KO MCF-7 cells were seeded in the six well plate and removed the medium before staining. Next, 1 mL cell culture medium and 1 mL JC-1 staining working solution were added. The cells were incubated for 20 min at 37 °C. After washing twice with JC-1 staining buffer, 2 mL cell culture medium was added, and the cells were observed under a laser confocal microscope (Nikon, Japan).

### Enzymatic removal of neuraminic acid

De-sialylation of breast cancer cells was achieved by incubating cells grown in a 96-well plate with 150 mU/mL neuraminidase (Cat.No. N3001, Sigma-Aldrich, USA) in DPBS for 6 h at 37 °C. Cells were then washed twice with DPBS and used for CCK-8 assay.

### CRISPR/Cas9 gene editing

MUC1 and GCNT3 was knocked out by CRISPR Cas9 gene editing system. The sequence of MUC1 and GCNT3 sgRNA were listed in Table S2. Cloning was performed using pLenti-CRISPR-V2 vector (Addgene, USA). The ligated vector was transformed into Trans Stbl3 Chemically Competent cells (Transgen, Beijing, China). Plasmid construction was performed according to protocol and confirmed by sequencing. The HEK 293 T cells were transfected with MUC1 knockout plasmids by Lipo8000™ Transfection Reagent (Cat.No. C0533, Beyotime, Shanghai, China) for 72 h to produce sufficient lentivirus. Cancer cells were seeded overnight and infected for 24 h. After 48 h, transfected cells were selected with 2 mg/ml puromycin, 300 ug/ml zeocin, 300 mg/ml G-418 or 2 ug/ml blasticidin for 1–2 week depending on antibiotic marker. Thereafter, single-cell cloning was amplified in 96-well plate. After growth, harvest the cells and confirm by western blot and qPCR.

### Lentivirus transfection

HEK 293 T cells were used to produce lentivirus. After cancer cells were seeded in dishes or plates and cultured overnight, the fresh medium containing 5 μg/ml polybrene (Cat.No. ST1380, Beyotime, Shanghai, China), collected lentivirus together with Lipo8000™ Transfection Reagent was used to culture cells for 24 h. Then cells were selected by indicated dose of puromycin or G-418 for a week to retain the successfully infected cells.

### Expression constructs

The WT MUC1 fragment was subcloned from the cDNA of MCF-7 and MDA-MB-231 cells then inserted into the pLenti-CMV-Neo vector (Addgene, USA). Transmembrane (TM) domain depleted mutant MUC1 (TMdel-mutant) was constructed with pLenti-CMV-Neo vector by Quik-Change Site-Directed PCR Mutagenesis Kit (Aligent, USA). The sequence for MUC1 transmembrane domain was obtained by reference [13] and Uniprot (https://www.uniprot.org) and the targeted sequence is: WGIALLVLVCVLVALAIVYLIAL. To construct O-glycosylation depleted mutant (GlyMut), all sites of Ser/Thr on N-terminal of MUC1 were mutated into Ala then cloned into pLenti-CMV-Neo vector. To construct O-glycosylated overexpression MUC1, fifteen tandem repeats (TR) were cloned into pLenti-CMV-MUC1flag-Neo vector subsequently after signal peptide (15TR_MUC1). Additionally, irregular O-glycosylation sequence named USTR was cloned likewise (USTR_MUC1). The sequence of MUC1-CT was deleted and replaced with GFP tag (CT_Repalce). The CQC motif of MUC1-CT was replaced with AQA in 15TR_MUC1 and GlyMut plasmids, respectively. The above fragments were constructed by GENEWIZ Company (Nanjing, China) and all plasmids were verified by Sanger sequencing. The sequences of 5TR, TM, USTR, and GlyMut were displayed in Table S2.

### Fluorescence density of intracellular apigenin

Cells were treated with apigenin (50 μM) or DMSO for 3 h at 37 °C in supplemented RPMI-1640 (without phenol red). Nuclear staining was done with 1 μg/mL DAPI (Cat.No. C0060, Solarbio, Beijing, China) for 15 min at 37 °C in dark. Cells were washed five times with PBS and stained with 0.1% (w/v) diphenylboric acid 2-aminoethyl ester (DPBA, Sigma; excitation 490 nm, emission 530 nm) for 1–2 min as previously described [56]. Fluorescence was visualized and photographed under laser confocal microscopy (Nikon, Japan).

### Sample preparation for ultra-high performance liquid chromatography (UPLC)

Seed the same number of cells in 10 cm dish. When the density was up to 90%, the culture medium containing 100 mM apigenin was replaced for 4 h rapid treatment. Remove the drug medium and completely wash the cells with cold PBS for 8–10 times, then quickly scrape off the cells with clean scraper and collect cells in the tube centrifuged at 13,000 × g for 30 min at 4 °C. To assess the flavone content, collected cells were extracted twice with equal volume of methanol centrifuged at 3000 × g, and the pellet extracted with 70% methanol. The supernatants were dried under N_2_ and reconstituted in methanol. Prior to UPLC analysis, filter the collected supernatant with 0.22 μm membrane.

### Construction of standard curves and chromatographic UPLC conditions

Standard apigenin (Selleck, USA) was dissolved in methanol solution with the concentration decreasing from 100 mg/L to 1 mg/L, establishing for a calibration curve inclusive of all concentrations of the apigenin samples (*y* = 21579x-59180, R² = 0.9959). The samples were analyzed by UPLC using an Acquity UPLC BEH C18 (100 × 2.1 mm, 1.7 μm) column and PDA detector (Waters Corp, Milford, USA). The mobile phases were 0.1% formic acid in water (A) and methanol (B). The gradient was set as follows: 0–3 min, 10% B; 3–7 min, 10% B; 7–13 min, 10–22% B; 13–22 min, 22 % B; 22–26 min, 22–28 % B; 26–33 min, 28–46 % B; 33–36 min, 46–10% B; 36–38 min, 10% B. The column temperature was maintained at 30 °C with a flow rate 0.2 mL/min. The sample temperature remained 18 °C. The injection volume was 1 µL.

### RNA-Seq analysis

Total RNA was extracted from WT and MUC1 KO MCF-7 cells. The mRNA was isolated with Oligo Magnetic Beads and randomly interrupted using divalent cation in NEB Fragmentation Buffer for cDNA synthesis. Libraries were generated using the NEB Next^®^ Ultra™ RNA Library Prep Kit for Illumina® (New England BioLabs, Ipswich, MA, USA) following the manufacturer’s instructions. Sequencing was conducted using the Illumina NovaSeq 6000 platform.

### Analysis of MUC1 expression level in multiple cancers

The expression level of the MUC1 gene in multiple types of cancers was identified in the Oncomine database (https://www.oncomine.org/resource/login.html) and GEPIA (http://gepia.cancer-pku.cn/index.html). The MUC1 expression level in patient tissue was detected by HPA (https://www.proteinatlas.org).

### Analysis of prognosis of MUC1 in breast cancer

The association between MUC1 expression and outcomes in patients with breast cancer was explored in PrognoScan Database. The hazard ratio (HR) with 95% confidence intervals and log-rank P-value were also computed.

### Drug sensitivity analysis

The correlation between gene expression and drug sensitivity was investigated in GSCA database (http://bioinfo.life.hust.edu.cn/web/GSCALite/). Drug sensitivity and gene expression profiling data of cancer cell lines in GDSC and CTRP are integrated for investigation. The expression of each gene in the gene set was performed by Spearman correlation analysis with the small molecule drug sensitivity (IC_50_).

### Tumor xenograft model

All animal experiments were approved by institutional animal care and used committees of China Agricultural University. Female BALB/c Nude mice were purchased from SPF Biotechnology Co., Ltd (Charles River, Beijing, China), and raised in the SPF animal center of China Agricultural University. The MCF-7 WT and MUC1 KO at a concentration of 1 × 10^6^ cells/100 μl mixture (PBS:Matrigel = 1:1, Gibco, Invitrogen, Carlsbad, CA, USA) each mice were inoculated subcutaneously on the right armpit of nude mice aged 5 weeks. After 1 week, the nude mice with successful xenogeneic tumor transplantation were randomly divided into four groups (eight mice per group), then administered with 40 mg/kg/day (intraperitoneal injection, i.p.) of apigenin for 16 days. The body weight of nude mice, tumor volume, and weight were measured.

### Statistical analysis

Experimental data are presented as mean ± S.D. from three independent experiments where applicable. In order to determine statistical probabilities, unpaired Student’s *t*-test or one-way ANOVA is used where appropriate. GraphPad Prism 8 software (GraphPad Prism 8.0, La Jolla, CA, USA), Image J and FlowJo were used for statistical analysis. The calculation of apigenin peak area of UPLC was measured by Empower software. *p < 0.05, **p < 0.01, ***p < 0.001, ****p < 0.0001. ns indicates no significant difference.

## Supplementary information

Table S1

Table S2

Supplementary figure 1

Supplementary figure 2

Supplementary figure 3

Supplementary figure 4

Supplementary figure 5

Supplementary figure 6

Supplementary figure 7

Supplementary figure 8

Supplementary figure 9

Original WB Data

aj-checklist

Supplementary figure legends

## Data Availability

The datasets used and analyzed during the current study are available from the corresponding author on reasonable request.
