# Supplementary material for: Glycosylated modification of MUC1 maybe a new target to promote drug sensitivity and efficacy for breast cancer chemotherapy
Source: Cell Death Dis. 2022 Aug 16;13(8):708. doi: 10.1038/s41419-022-05110-2 (PMC9378678; doi:10.1038/s41419-022-05110-2)
Supplement: Supplementary file 1 — Table S1 [file 41419_2022_5110_MOESM1_ESM.docx]

| **Name** | **Source** | **Cat No.** |
| --- | --- | --- |
| Apigenin | CHENGDU MUST, Chengdu, China | A0113 |
| Chrysin | CHENGDU MUST, Chengdu, China | A0292 |
| Diosmetin | CHENGDU MUST, Chengdu, China | A0927 |
| Luteolin | CHENGDU MUST, Chengdu, China | A0108 |
| Quercetin | CHENGDU MUST, Chengdu, China | A0083 |
| GO203 | Selleck, USA | S8674 |
| Benzyl-α-GalNAc (Benzyl 2-acetamido-2-deoxy-α-D-galactopyranoside) ≥97% (TLC) | Sigma, USA | B4894 |
| Tunicamycin | Abcam, UK | 11089-65-9 |
| Neuraminidase from Clostridium perfringens (Purified) | Aladdin, Shanghai, China | N128387-25UN |
| MUC1 (D9O8K) XP® Rabbit mAb | CST, USA | #14161 |
| Flag  (DYKDDDDK Tag (D6W5B) Rabbit mAb) | CST, USA | #14793 |
| HA Tag Monoclonal Antibody | ProteinTech, Wuhan, China | 66006-2-Ig |
| MYC Tag Monoclonal Antibody | ProteinTech, Wuhan, China | 60003-2-Ig |
| GCNT3 Rabbit pAb | ABclonal, Wuhan, China | A13209 |
| Lamin B1 Polyclonal Antibody | ProteinTech, Wuhan, China | 12987-1-AP |
| PARP Monoclonal Antibody | Beyotime,Shanghai, China | AP102 |
| Cleaved-PARP Monoclonal Antibody | Beyotime, Shanghai, China | AF1567 |
| Histone H3 Monoclonal Antibody | Beyotime, Shanghai, China | AF0009 |
| β-actin Antibody | ProteinTech, Wuhan, China | 20536-1-AP |
| Heochst 33342 | Beyotime, Shanghai, China | C1022 |
| Phospho-Histone H2AX (Ser139) Rabbit Polyclonal Antibody | Beyotime, Shanghai, China | AF5836 |
| MDR-1 (P glycoprotein Polyclonal antibody) | ProteinTech, Wuhan, China | 22336-1-AP |
| MRP1, ABCC1 Monoclonal Antibody | ProteinTech, Wuhan, China | 67228-1-Ig |
| Cyclin A2 Rabbit Monoclonal Antibody | Beyotime, Shanghai, China | AF2524 |
| HRP-labeled Goat Anti-Rabbit IgG  HRP-labeled Goat Anti-Mouse IgG | Beyotime, Shanghai, China  Beyotime, Shanghai, China | A0208  A0216 |
|  |  |  |
