# Supplementary material for: Glycosylated modification of MUC1 maybe a new target to promote drug sensitivity and efficacy for breast cancer chemotherapy
Source: Cell Death Dis. 2022 Aug 16;13(8):708. doi: 10.1038/s41419-022-05110-2 (PMC9378678; doi:10.1038/s41419-022-05110-2)
Supplement: Supplementary file 2 — Table S2 [file 41419_2022_5110_MOESM2_ESM.docx]

| GENE NAME | Accession number | F-primer (5’-3’) | R-primer (5’-3’) |
| --- | --- | --- | --- |
| MUC1 | NM_002456.6 | GTTACGGGTTCTGGTCATGC | TAGTCGGTGCTGGGATCTTC |
| NDUFA1 | NM_004541.4 | ATGTGGTTCGAGATTCTCCCC | CCTGTGGATGTACGCAGTAGC |
| NDUFB8 | NM_005004.4 | CCGCCAAGAAGTATAATATGCGT | TATCCACACGGTTCCTGTTGT |
| NDUFA11 | NM_175614.5 | GCCGAAGGTTTTTCGTCAGTA | GGAGGATTGAGTGTGACTCTGT |
| NDUFA2 | NM_002488.5 | GCAGCAAGTCGAGGAGTCG | CGTTTCTCAATGAAGTCCCTGA |
| NDUFB7 | NM_004146.6 | GGTAGGAGCTAGGTGACCCT | GTAGTCTGGCGGGAAGGTTG |
| NDUFA9 | NM_005002.5 | GTCACGTTCTGCCATTACTGC | GGTGGTTGACAACATATCGCC |
| COX8A | NM_004074.3 | GCCAAGATCCATTCGTTGCC | CTCTGGCCTCCTGTAGGTCT |
| COX6A1 | NM_004373.4 | AGTTGGTGTGTCCTCGGTTTC | GTGAGAGTCTTCCACATGCGA |
| COX5B | NM_001862.3 | ATGGCTTCAAGGTTACTTCGC | CCCTTTGGGGCCAGTACATT |
| COX6B1 | NM_001863.5 | CTACAAGACCGCCCCTTTTGA | GCAGAGGGACTGGTACACAC |
| ATP5ME | NM_007100.4 | CAGGTCTCTCCGCTCATCAAG | GCCCGAGGTTTTAGGTAATTGT |
| ATP5F1D | NM_001687.5 | ACTCTTCGGTGCAGTTGTTGG | GCCTCGATTCGGATCTGGAT |
| ATP5F1E | NM_006886.4 | GGCTGGACTCAGCTACATCC | TTACGTTGCTGCCAGAAGTCT |
| ATP5MG | NM_006476.5 | ATGGCCCAATTTGTCCGTAAC | TGGCGTAGTACCAAAATGTGG |
| GAPDH | NM_002046.7 | TTCCACCCATGGCAAATTCC | TCATGGTTCACACCCATGAC |
| ACTB | NM_001101.5 | GTCTCCTCTGACTTCAACAGCG | ACCACCCTGTTGCTGTAGCCAA |
|  |  |  |  |

| GENE | F-primer (5’-3’) | R-primer (5’-3’) |
| --- | --- | --- |
| human MUC1 sg1 | CACCGCAGCAGGAAGAAAGGAGAC | AAACGTCTCCTTTCTTCCTGCTGC |
| human MUC1 sg2 | CACCGACTGGGTGCCCGGTGTCA | AAACTGACACCGGGCACCCAGTC |
| human MUC1 sg3 | CACCGCAGGAAGAAAGGAGACT | AAACAGTCTCCTTTCTTCCTGC |
| humanGCNT3 sg1 | CACCGGAAGTGTGACTCTGACCACT | AAACAGTGGTCAGAGTCACACTTCC |
| humanGCNT3 sg2 | CACCGGGCTATTCTGAATAACCTGG | AAACCCAGGTTATTCAGAATAGCCC |
| humanGCNT3 sg3 | CACCGGCAAGCTGACCTCAACTGCA | AAACTGCAGTTGAGGTCAGCTTGCC |
|  |  |  |

5TR Sequence: GGGAGCACAGCACCGCCAGCCCATGGAGTGACGTCCGCGCCTGACACAAGGCCTGCACCAGGTAGTACAGCGCCACCGGCGCACGGCGTCACTTCTGCGCCAGATACCCGACCAGCCCCTGGCTCAACCGCTCCCCCTGCCCACGGAGTTACCTCAGCTCCCGACACTAGGCCAGCTCCTGGCAGCACTGCCCCTCCCGCGCATGGCGTGACCAGCGCCCCAGACACCCGGCCTGCTCCAGGATCCACCGCCCCACCCGCTCACGGGGTAACCTCCGCTCCGGATACGAGACCGGCGCCA

USTR Sequence:

ACCACAGCCCCTAAACCCGCAACAGTTGTTACGGGTTCTGGTCATGCAAGCTCTACCCCAGGTGGAGAAAAGGAGACTTCGGCTACCCAGAGAAGTTCAGTGCCCAGCTCTACTGAGAAGAATGCTGTGAGTATGACCAGCAGCGTACTCTCCAGCCACAGCCCCGGTTCAGGCTCCTCCACCACTCAGGGACAGGATGTCACTCTGGCCCCGGCCACGGAACCAGCTTCAGGTTCAGCTGCCACCTGGGGA

TM Sequence:

TGGGGCATCGCGCTGCTGGTGCTGGTCTGTGTTCTGGTTGCGCTGGCCATTGTCTATCTCATTGCCTTG

GlyMut Sequence:

ATGACACCGGGCACCCAGTCTCCTTTCTTCCTGCTGCTGCTCCTCACAGTGCTTACAGTTGTTACAGGTGCCGGTCATGCAGCCGCTGCCCCAGGTGGAGAAAAGGAGGCCGCCGCTGCCCAGAGAGCCGCCGTGCCCGCCGCTGCCGAGAAGAATGCTTTTAATGCCGCCCTGGAAGATCCCGCCGCCGACTACTACCAAGAGCTGCAGAGAGACATTGCCGAAATGTTTTTGCAGATTTATAAACAAGGGGGTTTTCTGGGCCTCGCCAATATTAAGTTCAGGCCAGGAGCCGTGGTGGTACAATTGGCCCTGGCCTTCCGAGAAGGTGCCATCAATGTCCACGACATGGAGGCCCAGTTCAATCAGTATAAAGCCGAAGCAGCCGCCCGATATAACCTGGCCATCGCCGACGTCGCCGTGGCCGATGTGCCATTTCCTTTCGCCGCCCAGGCCGGGGCTGGGGTGCCAGGCTGGGGCATCGCGCTGCTGGTGCTGGTCTGTGTTCTGGTTGCGCTGGCCATTGTCTATCTCATTGCCTTGGCTGTCTGTCAGTGCCGCCGAAAGAACTACGGGCAGCTGGACATCTTTCCAGCCCGGGATACCTACCATCCTATGAGCGAGTACCCCACCTACCACACCCATGGGCGCTATGTGCCCCCTAGCAGTACCGATCGTAGCCCCTATGAGAAGGTTTCTGCAGGTAATGGTGGCAGCAGCCTCTCTTACACAAACCCAGCAGTGGCAGCCACTTCTGCCAACTTGTGA
