# Supplementary figures and images for: Glycosylated modification of MUC1 maybe a new target to promote drug sensitivity and efficacy for breast cancer chemotherapy
Source: Cell Death Dis. 2022 Aug 16;13(8):708. doi: 10.1038/s41419-022-05110-2 (PMC9378678; doi:10.1038/s41419-022-05110-2)

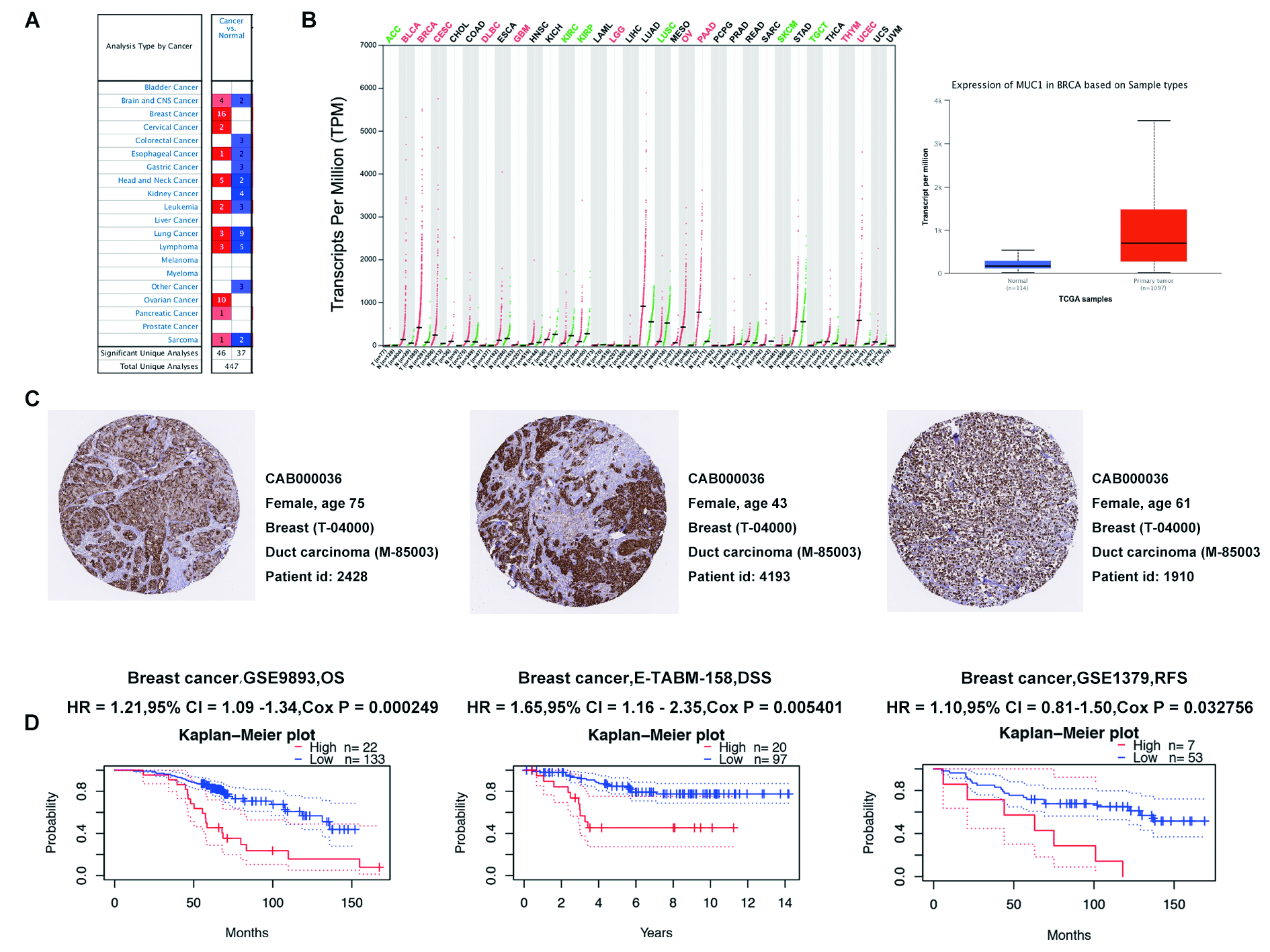

Supplement: Supplementary file 3 — Supplementary figure 1 [file 41419_2022_5110_MOESM3_ESM.tif]

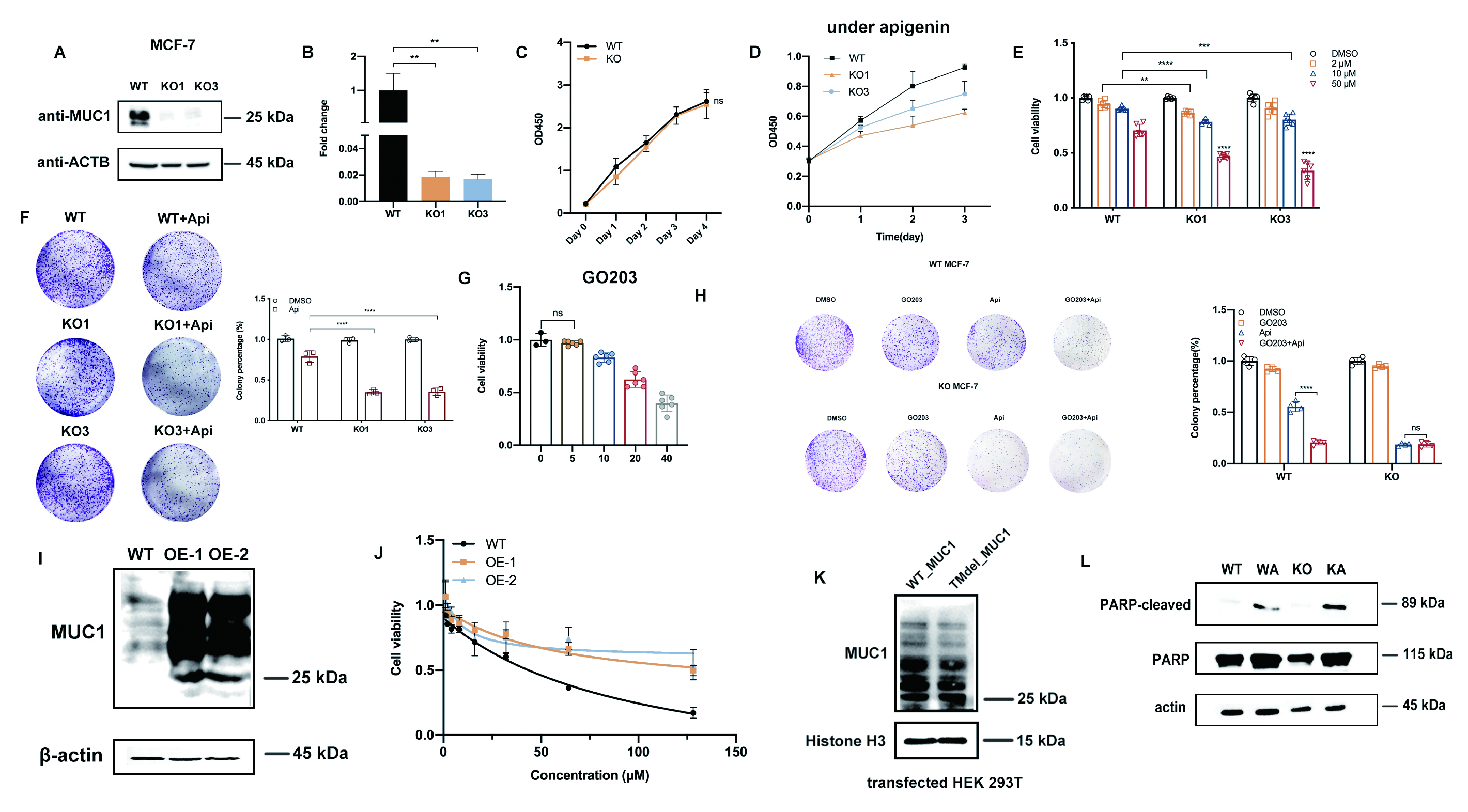

Supplement: Supplementary file 4 — Supplementary figure 2 [file 41419_2022_5110_MOESM4_ESM.tif]

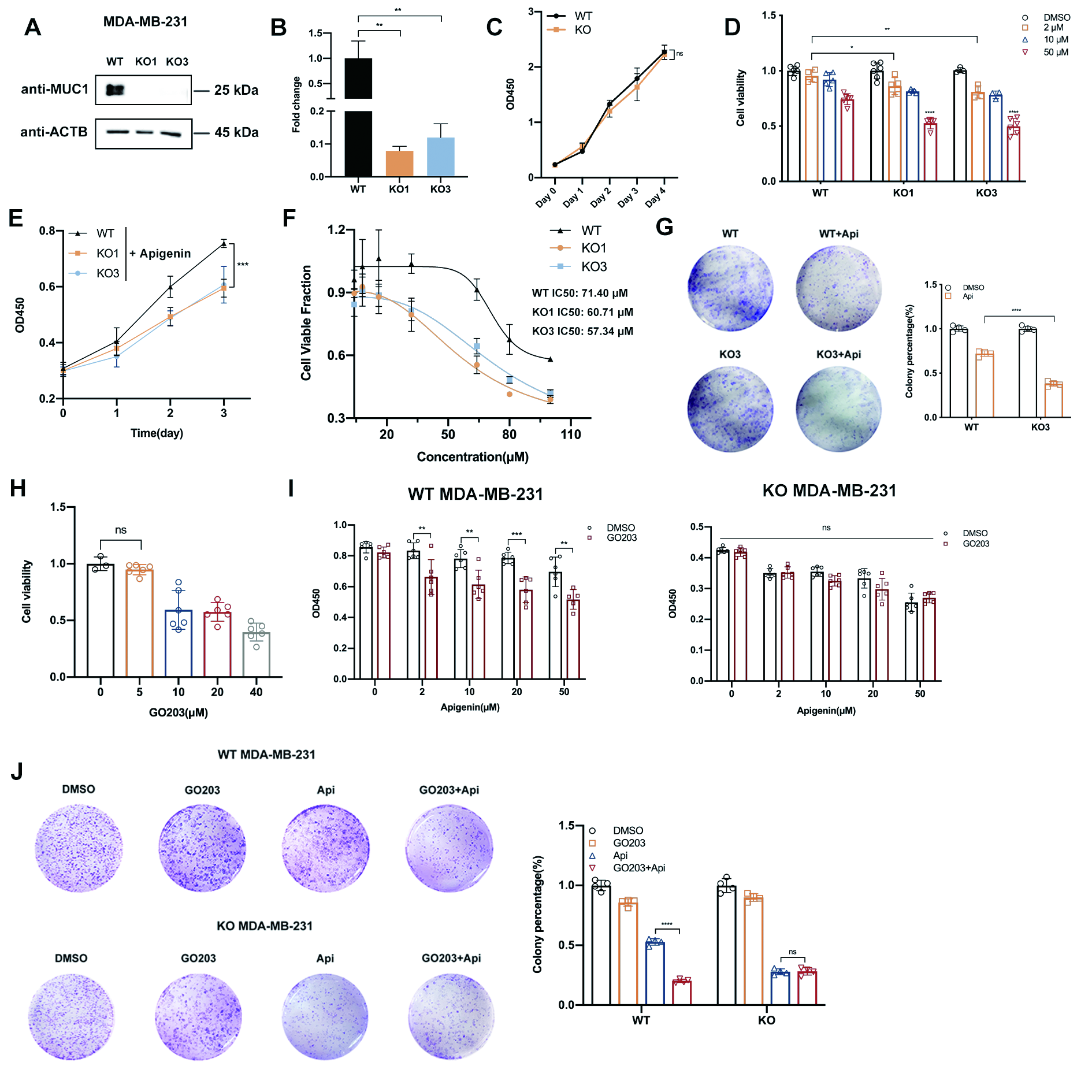

Supplement: Supplementary file 5 — Supplementary figure 3 [file 41419_2022_5110_MOESM5_ESM.tif]

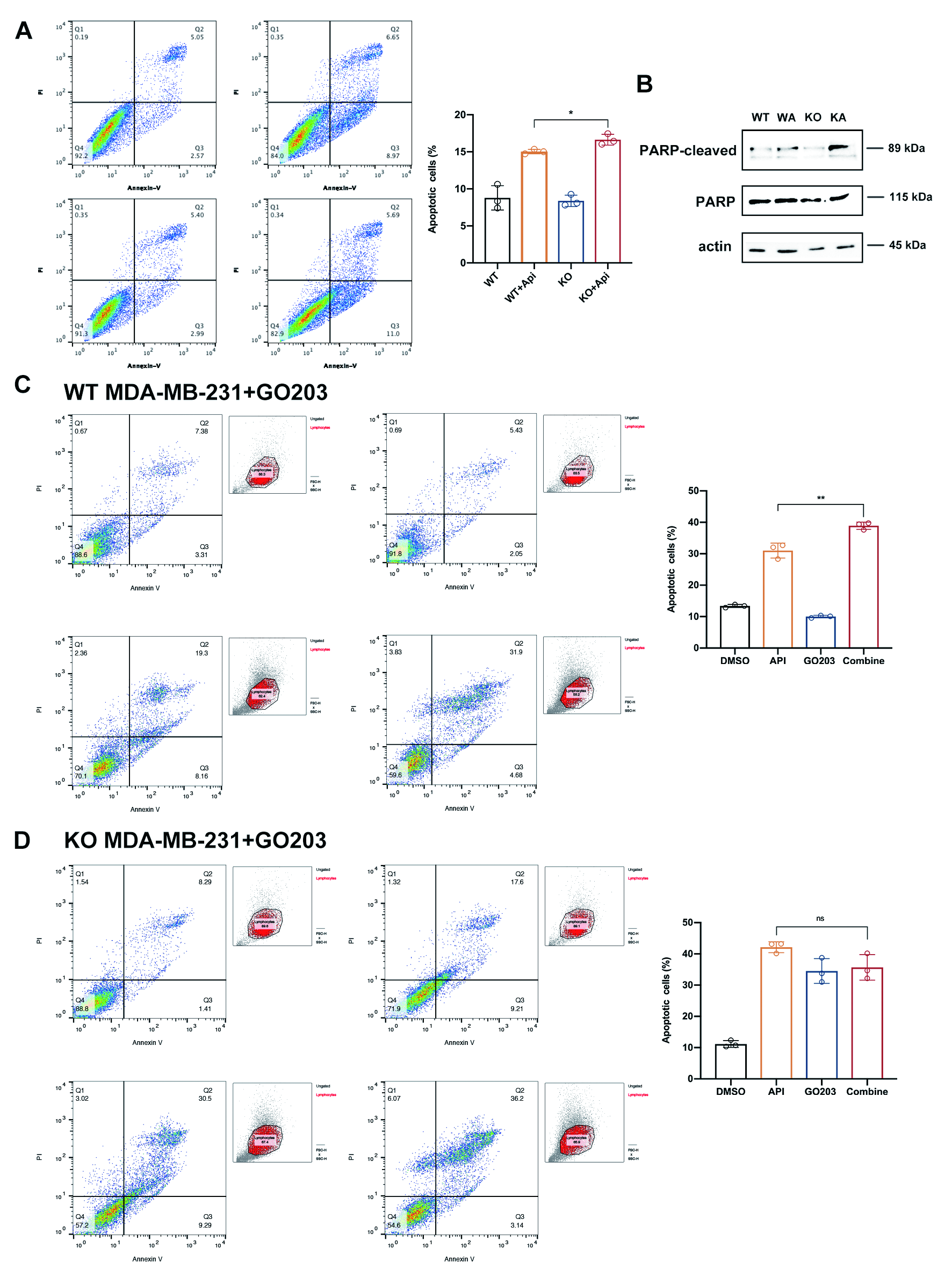

Supplement: Supplementary file 6 — Supplementary figure 4 [file 41419_2022_5110_MOESM6_ESM.tif]

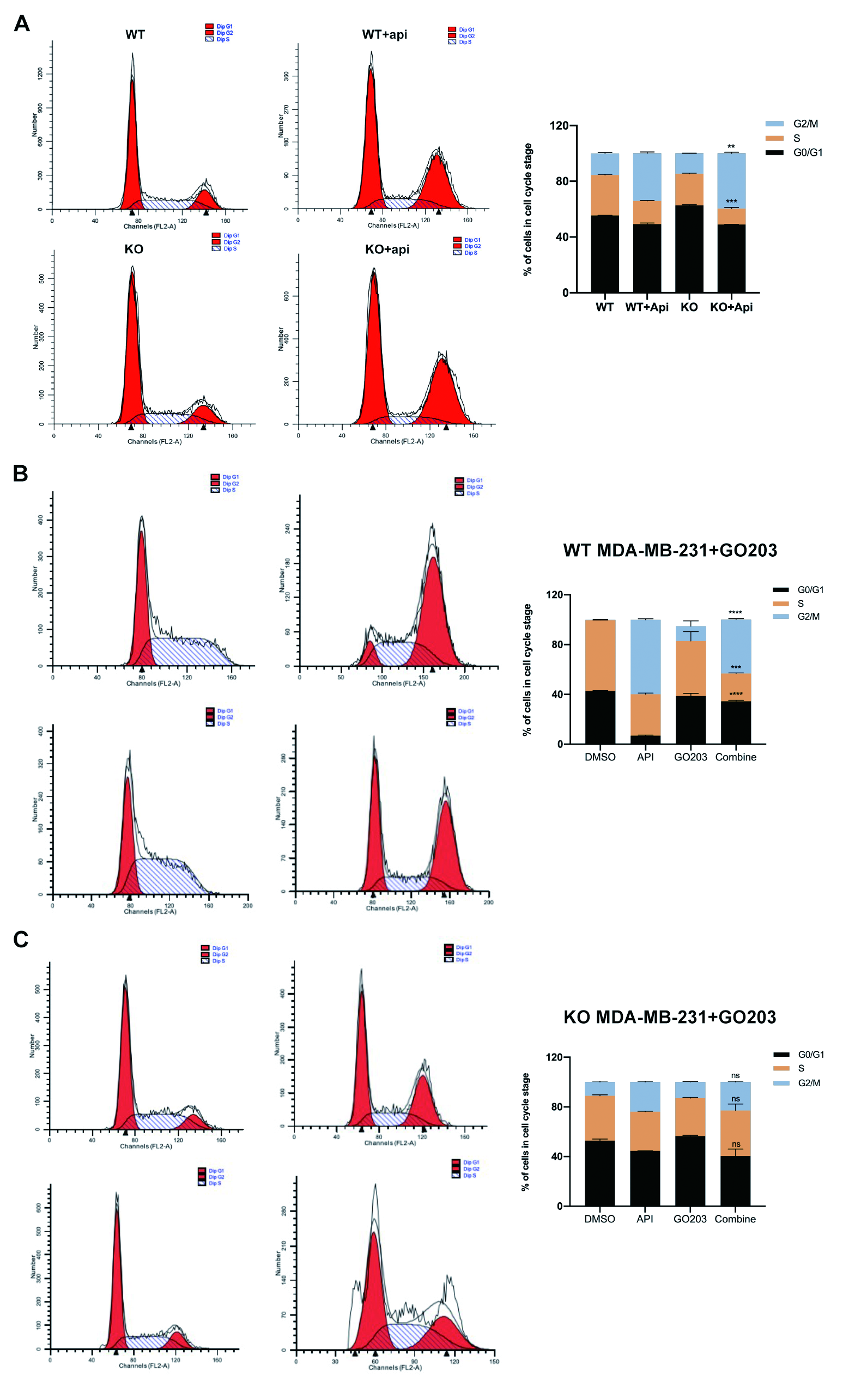

Supplement: Supplementary file 7 — Supplementary figure 5 [file 41419_2022_5110_MOESM7_ESM.tif]

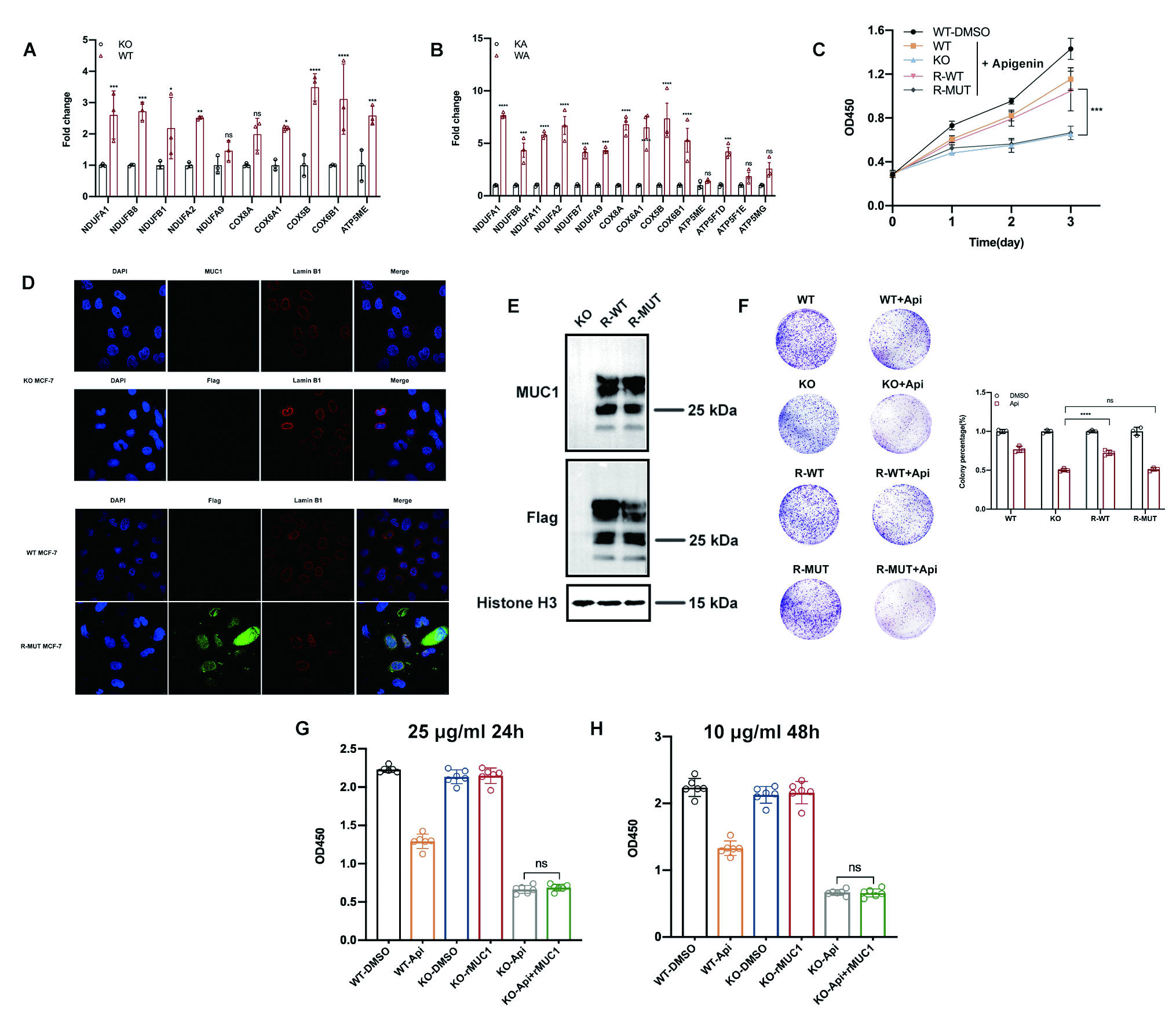

Supplement: Supplementary file 8 — Supplementary figure 6 [file 41419_2022_5110_MOESM8_ESM.tif]

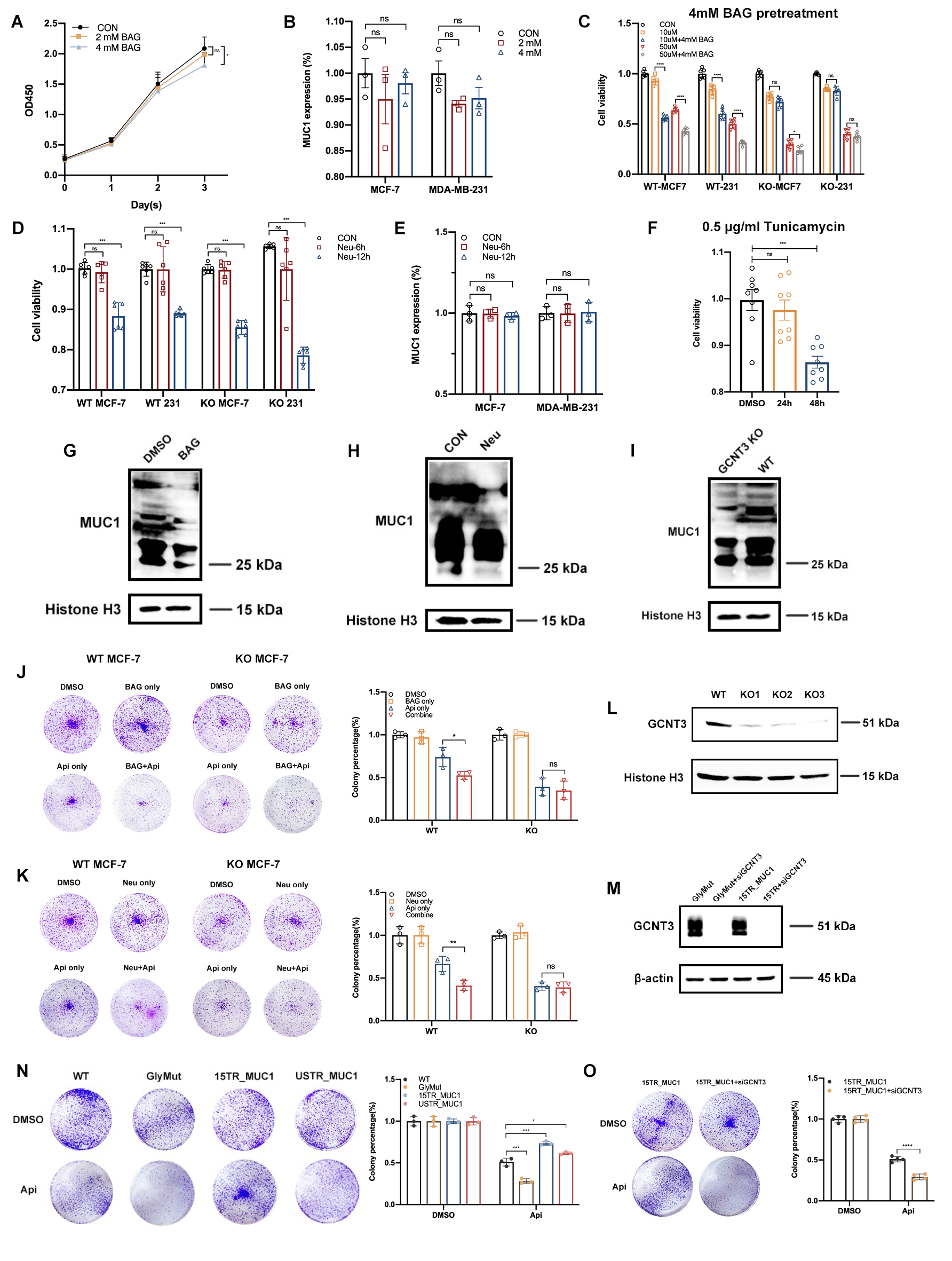

Supplement: Supplementary file 9 — Supplementary figure 7 [file 41419_2022_5110_MOESM9_ESM.tif]

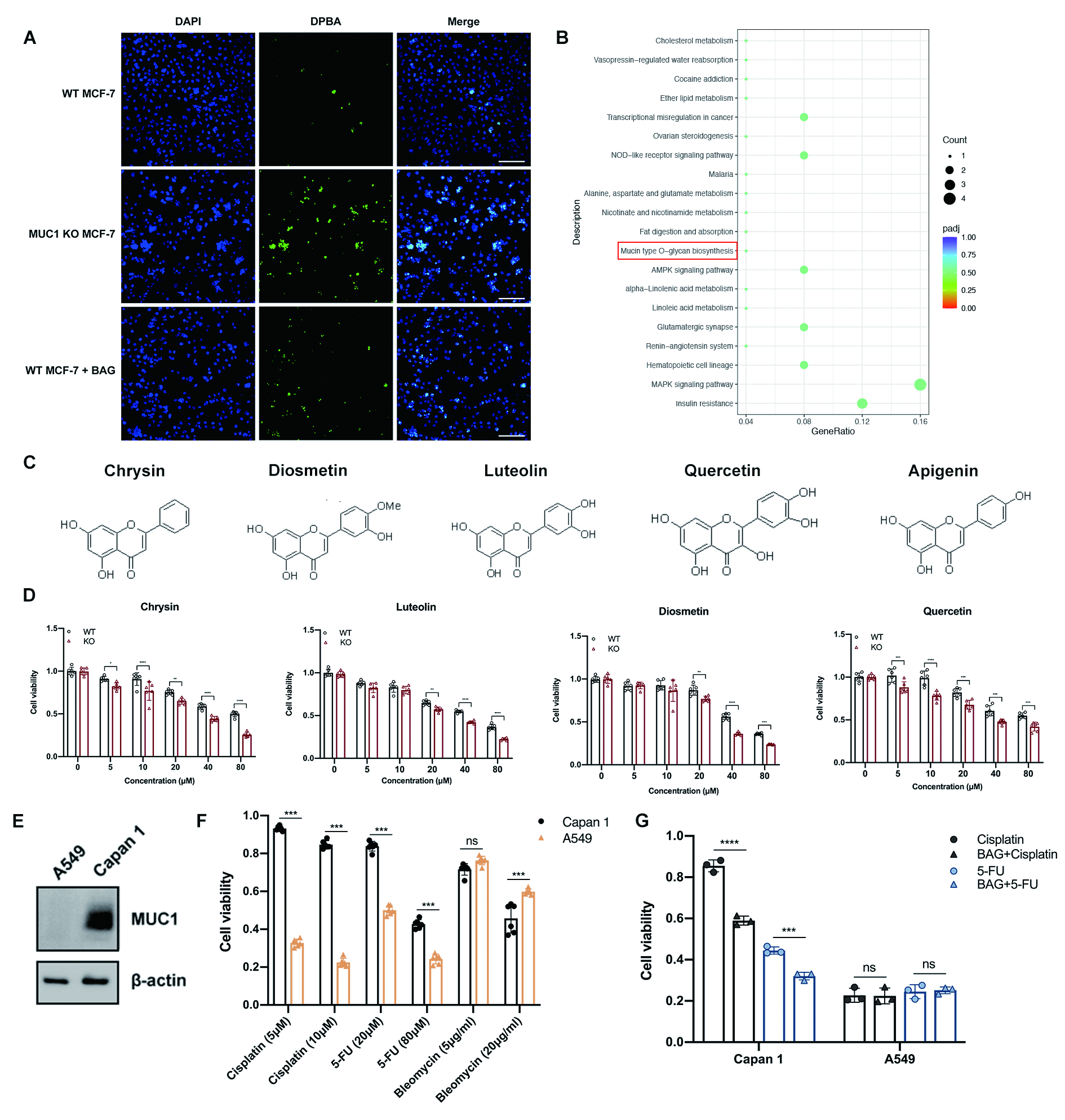

Supplement: Supplementary file 10 — Supplementary figure 8 [file 41419_2022_5110_MOESM10_ESM.tif]

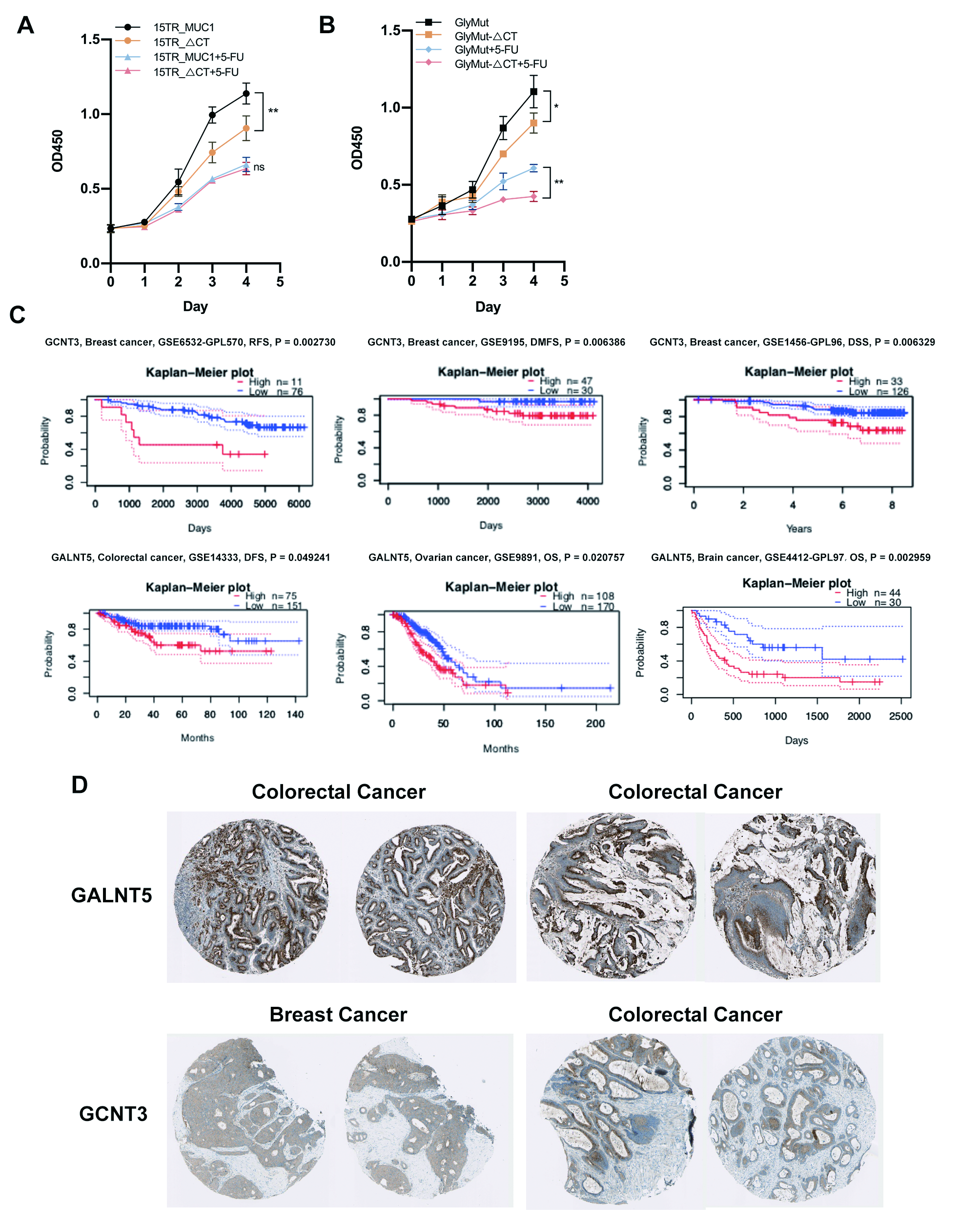

Supplement: Supplementary file 11 — Supplementary figure 9 [file 41419_2022_5110_MOESM11_ESM.tif]
