# Supplementary material for: Glycosylated modification of MUC1 maybe a new target to promote drug sensitivity and efficacy for breast cancer chemotherapy
Source: Cell Death Dis. 2022 Aug 16;13(8):708. doi: 10.1038/s41419-022-05110-2 (PMC9378678; doi:10.1038/s41419-022-05110-2)
Supplement: Supplementary file 12 — Original WB Data [file 41419_2022_5110_MOESM12_ESM.pdf]

Figure 4F

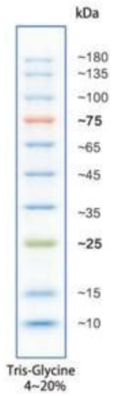

used protein marker

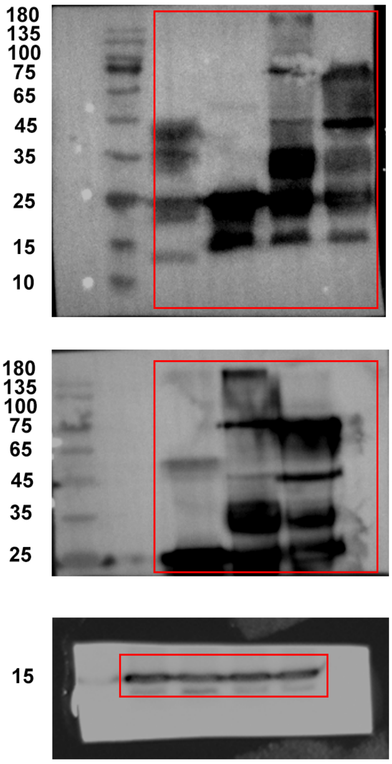

anti-MUC1 25-180 kD

anti-Flag 25-180 kD

anti-Histone H3 15kD

Figure S2A

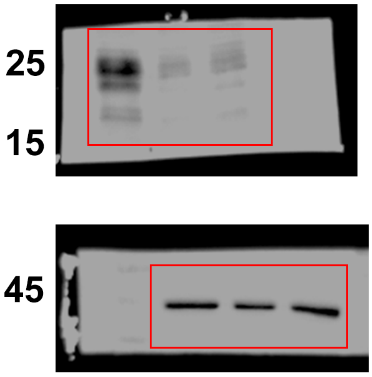

anti-MUC1 25kD

anti-actin 42kD

Figure S2I

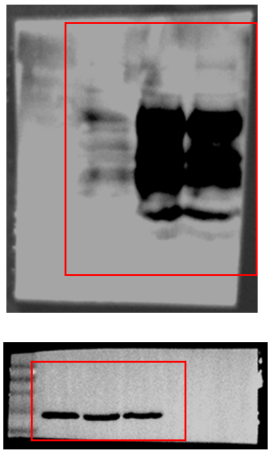

Figure S2K

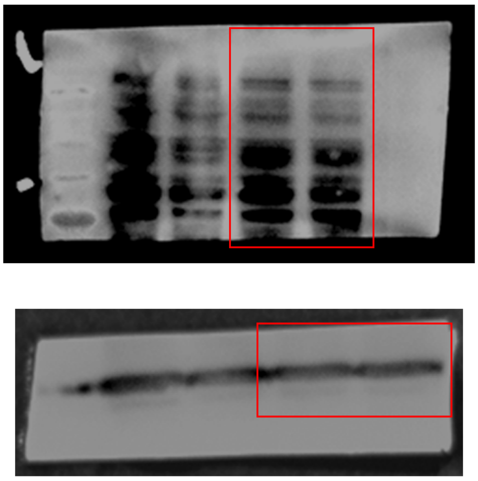

Figure S2L

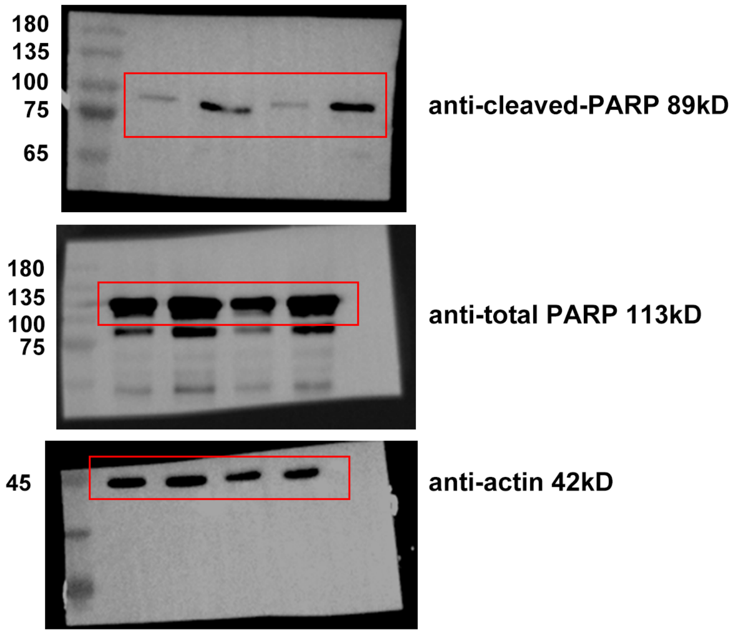

Figure S3A

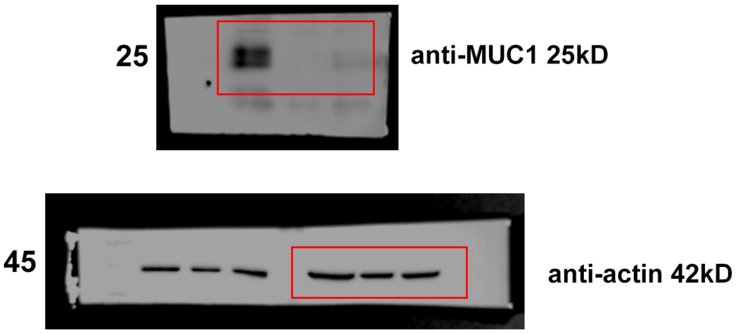

Figure S6E

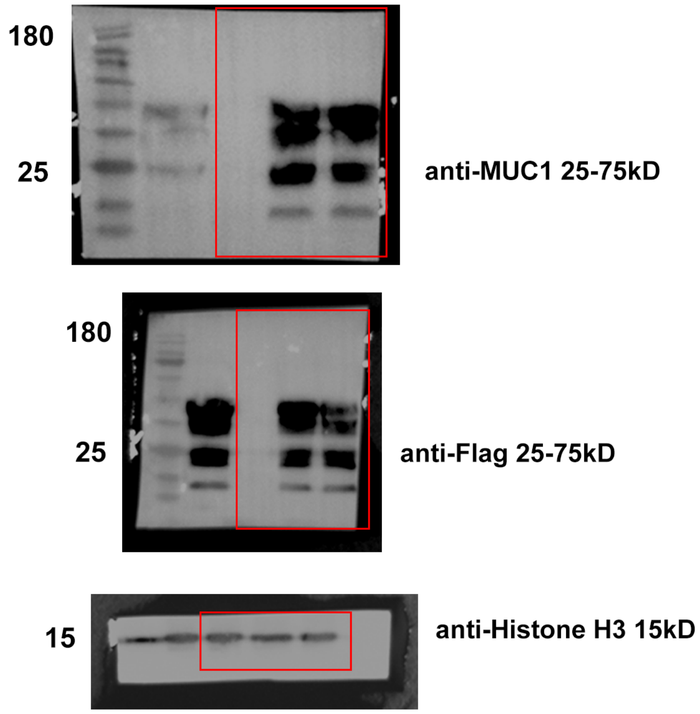

Figure S7G

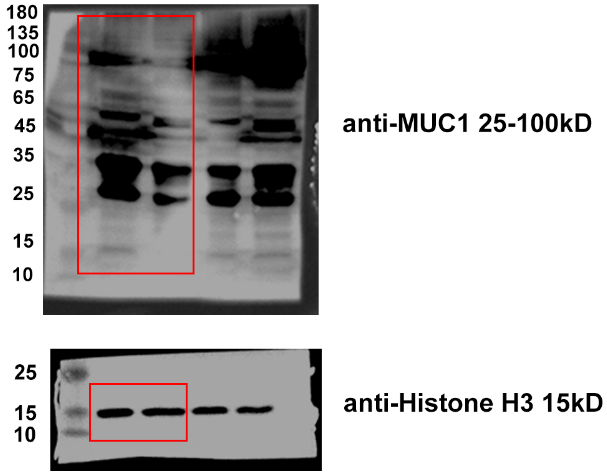

Figure S7H

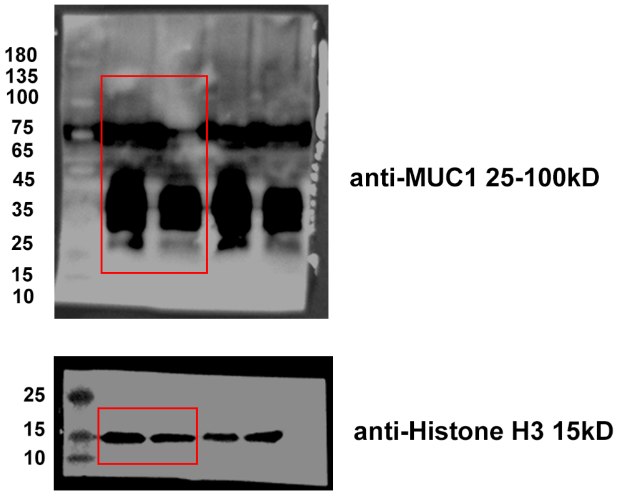

Figure S7I

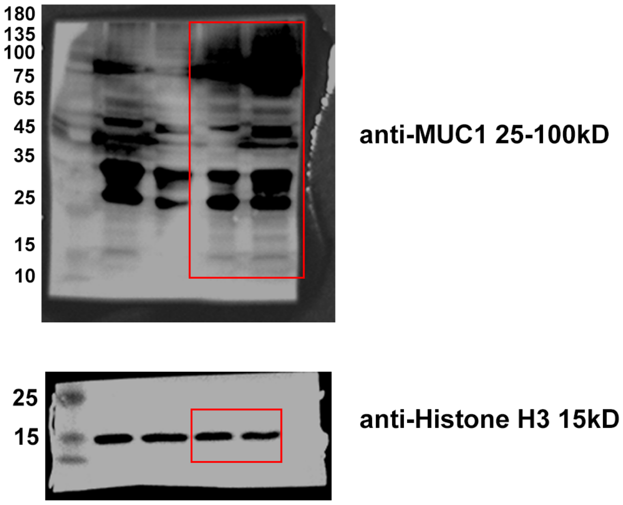

Figure S7L

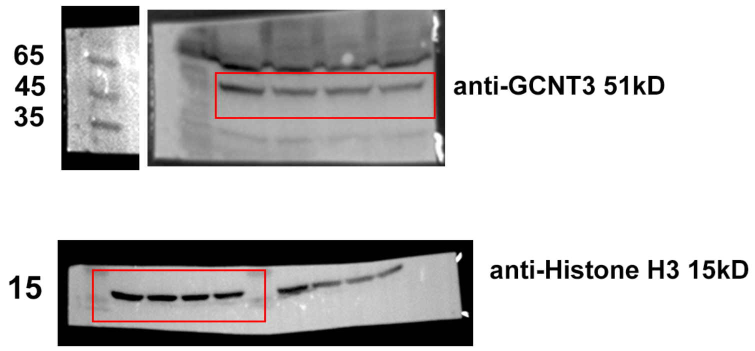

Figure S7M

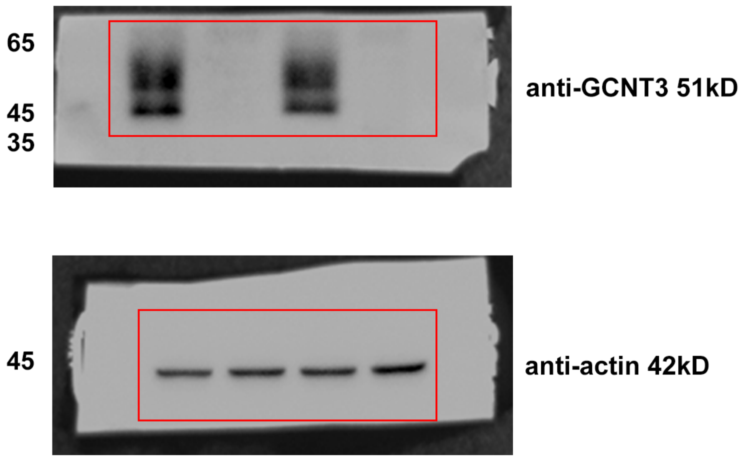

Figure S8E

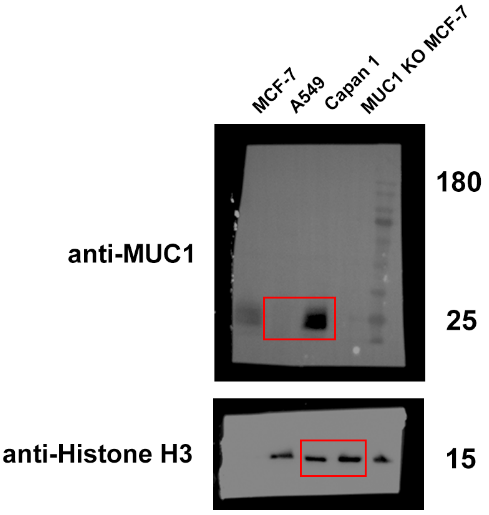
MCF-7
A549
Capan 1
MUC1 KO MCF-7
anti-MUC1
180
25
anti-Histone H3
15
