# Supplementary material for: Glycosylated modification of MUC1 maybe a new target to promote drug sensitivity and efficacy for breast cancer chemotherapy
Source: Cell Death Dis. 2022 Aug 16;13(8):708. doi: 10.1038/s41419-022-05110-2 (PMC9378678; doi:10.1038/s41419-022-05110-2)
Supplement: Supplementary file 14 — Supplementary figure legends [file 41419_2022_5110_MOESM14_ESM.docx]

**Supplementary Figure 1: MUC1 was overexpressed in breast cancer and correlated with worse prognosis in patients**

**A** MUC1 expression in pan-cancer from Oncomine database. **B** Compared with normal tissue, MUC1 is highly expressed in breast cancer tumor. **C** High level of MUC1 is found in breast cancer tissues. **D** High level of MUC1 indicates worse prognosis in breast cancer patients.

**Supplementary Figure 2:** **Inhibiting MUC1 enhanced the cytotoxic sensitivity to apigenin in MCF-7 breast cancer cells.**

**A** Western blot verification of KO efficiency. **B** qPCR analysis of WT/KO cells. **C** Continuous growth of WT/KO cells for four days and there was no significant difference. **D** Growth rate of WT/KO cells under 30 μM apigenin for three days. **E** Cell viability of MCF-7 WT/KO cells treated with indicated dose of apigenin. **F** Colony formation of WT/KO cells treated with 25 μM apigenin for a week. **G** The confirmation for GO203 cytotoxicity. **H** Colony formation of WT/KO cells pretreated with 5 μM GO203 thereafter 25 μM apigenin for one week. **I** Western blot verification of MUC1 overexpression in MCF-7 cells. **J** IC_50_ of WT and OE cells treated with various doses of apigenin. **K** Western blot verification of MUC1 in HEK 293T cells transfected with WT_MUC1 or TMdel_MUC1 plasmid. **L** Western blot verification of PARP and cleaved-PARP in WT/KO cells treated with apigenin. Each group was analyzed in triplicate. *P<0.05; **P<0.01; ***P<0.001 for comparisons.

**Supplementary Figure 3: Suppressing MUC1 elevated the drug sensitivity and efficacy in MDA-MB-231 cells.**

**A** Western blot verification of KO efficiency in MDA-MB-231 cells. **B** qPCR analysis of MUC1 in WT or MUC1 KO MDA-MB-231 cells. **C** Continuous growth of WT/KO cells for four days and no significant difference on cell growth was found. **D** Cell viability of WT/KO cells treated with apigenin for 48 h. **E** Cell viability of WT/KO cells treated with 30 μM apigenin for three days. **F** IC_50_ of WT/KO cells treated with apigenin for 48 h. **G** **Left**: Colony formation assay of WT/KO cells treated with 25 μM apigenin for a week. **Right:** The Quantiﬁcation of **G**. **H** Cell viability of MDA-MB-231 cells under different concentrations of GO203. **I** **Left:** Cell viability of WT MDA-MB-231 cells treated with 5 μM GO203 combined with different doses of apigenin. **Right:** Cell viability of KO MDA-MB-231 cells treated with 5 μM GO203 combined with different doses of apigenin. **J** **Left:** Colony formation of WT/KO cells pre-treated with GO203 thereafter 25 μM apigenin for one week. **Right:** The qualification of **J**. Each group was analyzed in triplicate. *P<0.05; **P<0.01; ***P<0.001 for comparisons.

**Supplementary Figure 4: Targeting MUC1 promoted the apigenin induced apoptosis in MDA-MB-231 cells**

**A** **Left**: Apoptotic ﬂow cytometry of WT/KO MDA-MB-231 cells treated with 50 μM apigenin for 48 h. **Right**: Quantiﬁcation of **A**. **B** Western blot verification of PARP and cleaved-PARP with/without apigenin treatment. **C** Apoptotic ﬂow cytometry of WT MDA-MB-231 cells treated with GO203 and apigenin for 48 h. **D** Apoptotic ﬂow cytometry of KO MDA-MB-231 cells treated with GO203 and apigenin for 48 h. Each group was analyzed in triplicate. *P<0.05; **P<0.01; ***P<0.001 for comparisons.

**Supplementary Figure 5: Targeting MUC1 prompted the apigenin induced cell cycle arrest in MDA-MB-231 cells**

**A** Cell cycle ﬂow cytometry of WT/KO MDA-MB-231 cells treated with 50 μM apigenin for 48 h. **B** Cell cycle ﬂow cytometry of WT MDA-MB-231 cells treated with GO203 and apigenin for 48 h. **C** Cell cycle ﬂow cytometry of KO MDA-MB-231 cells treated with GO203 and apigenin for 48 h. Each group was analyzed in triplicate. *P<0.05; **P<0.01; ***P<0.001 for comparisons.

**Supplementary Figure 6: Verification of RNA-Seq results and MUC1 subcellular localization by immunoﬂuorescence assay in MCF-7 cells .**

**A** The qPCR verification of genes in terms of mitochondria respiratory chain complexes in WT/KO cells. **B** The qPCR verification of genes in terms of mitochondria respiratory chain complexes in WT/KO cells treated with 50 μM apigenin for 48 h. **C** Cell viability of five cell lines under 25 μM apigenin. **D** Immunoﬂuorescence assay using MUC1 and Flag antibodies was performed to confirm the MUC1 and Flag distribution. Blue: Hoechst for nuclear staining; Red: Lamin B1 for nuclear membrane staining; Green: MUC1 or Flag staining. In KO cells, no MUC1 or Flag was seen. In WT cells, no flag was seen. In R-MUT cells, flag mostly assembled near the nuclear, in line with MUC1 distribution. **E** Western blot verification of MUC1 in WT/R-WT/R-MUT cell lines. **F** Colony formation of WT/R-WT/R-MUT cell lines treated with 25 μM apigenin for a week. **G** The OD value of the cells treated with apigenin and recombinant MUC1 (25 μg/ml) for 24 h. **H** The OD value of the cells treated with apigenin and recombinant MUC1 (10 μg/ml) for 48 h. Each group was analyzed in triplicate. *P<0.05; **P<0.01; ***P<0.001 for comparisons.

**Supplementary Figure 7: Inhibiting MUC1 glycosylation revived the chemosensitivity to drugs in MCF-7 cells.**

**A** The OD value of MCF-7 cells treated with DMSO, 2 mM and 4 mM BAG for three days. No extra harm was observed in 2 mM BAG treatment. **B** The qPCR qualification of MUC1 mRNA with BAG treatment for 24 h. No significant difference was observed. **C** Cell viability of WT/KO cells pretreated with 4 mM BAG post apigenin treatment. Both MCF-7 and MDA-MB-231 were detected. **D** Cell viability of WT/KO cells treated with neuraminidase for indicated time. Both MCF-7 and MDA-MB-231 were detected. **E** The qPCR qualification of MUC1 mRNA with neuraminidase treatment for indicated time. No significant difference was observed. **F** Cell viability of MCF-7 cells treated with 0.5 μg/ml tunicamycin for indicated time. **G** Western blot verification of MUC1 from BAG-treated cells. **H** Western blot verification of MUC1 from neuraminidase-treated cells. **I** Western blot verification of MUC1 from GCNT3 KO cells. **J** **Left:** Colony formation of WT/KO cells pretreated with BAG post 25 μM apigenin treatment for a week. **Right:** The quantiﬁcation of **J**. **K** **Left:** Colony formation of WT/KO cells pretreated with Neu post 25 μM apigenin treatment for a week. **Right:** The quantiﬁcation of **K**. **L** Western blot verification of GCNT3 KO. **M** Western blot verification of GCNT3 inhibition with siRNA **N** Colony formation of WT/GlyMut/15TR_MUC1/USTR_MUC1 MCF-7 cells treated with 25 μM apigenin for a week. **O** Colony formation of GlyMut/15TR_MUC1 MCF-7 cells transfected with GCNT3 siRNA then treated with 25 μM apigenin for one week. Each group was analyzed in triplicate. *P<0.05; **P<0.01; ***P<0.001 for comparisons.

**Supplementary Figure 8**

**A** The fluorescence distribution of intracellular apigenin mediated by DPBA staining. **B** The GO analysis of WT/KO MCF-7 cells (upregulated signaling pathways). Mucin-type O-glycan biosynthesis was elevated in KO group. **C** Chemical structure of five apigenin analogs. **D** Cell viability of WT/KO cells treated with five natural flavonoids, respectively. **E** Western blot verification of MUC1 expression in A549 and Capan 1 cell lines. **F** Cell viability of A549 or Capan 1 cells treated with indicated dose of cisplatin, 5-FU or bleomycin for 48 h. **G** Cell viability of Capan 1 cells pretreated with 2 mM BAG for 12 h thereafter cisplatin (10 μM) or 5-FU (80 μM) for 48 h. *P<0.05; **P<0.01; ***P<0.001 for comparisons.

**Supplementary Figure 9: High level of MUC1 and related glycosyltransferase predict worse survival in cancer patients.**

**A** OD 450 under continuous growth of cell lines as shown. 15TR_MUC1 was the plasmid that over-expressed O-glycosylation on MUC1-N. 15TR_△CT was the mutant of 15TR_MUC1 whose CQC was replaced with AQA. **B** OD 450 under continuous growth of cell lines as shown. GlyMut was the plasmid that lacks O-glycosylation (replace all Ser/Thr with Ala) sites on MUC1-N. GlyMut _△CT was the mutant of GlyMut whose CQC was replaced with AQA. All plasmids were transiently transfected into MUC1 KO MCF-7 cells. **C** Kaplan-Meier plots of GCNT3 or GALNT5 in cancer patients from GSE database. Patients with less expression of GCNT3 or GALNT5 predict better survival. **D** The expression of GALNT5 or GCNT3 in human cancer tissues from HPA database.
